# Causal Association of Type 2 Diabetes Mellitus and Glycemic Traits With Cardiovascular Diseases and Lipid Traits: A Mendelian Randomization Study

**DOI:** 10.3389/fendo.2022.840579

**Published:** 2022-04-22

**Authors:** Mingkai Huang, Loum-Davadi Laina-Nicaise, Lingfeng Zha, Tingting Tang, Xiang Cheng

**Affiliations:** ^1^ Department of Cardiology, Union Hospital, Tongji Medical College, Huazhong University of Science and Technology, Wuhan, China; ^2^ Key Laboratory of Biological Targeted Therapy of the Ministry of Education, Union Hospital, Tongji Medical College, Huazhong University of Science and Technology, Wuhan, China

**Keywords:** Mendelian randomization, diabetes, glycemic traits, cardiovascular disease, lipid

## Abstract

**Objective:**

We aimed to evaluate the causal effect of type 2 diabetes mellitus (T2DM) and glycemic traits on the risk of a wide range of cardiovascular diseases (CVDs) and lipid traits using Mendelian randomization (MR).

**Methods:**

Genetic variants associated with T2DM, fasting glucose, fasting insulin, and hemoglobin A1c were selected as instrumental variables to perform both univariable and multivariable MR analyses.

**Results:**

In univariable MR, genetically predicted T2DM was associated with higher odds of peripheral artery disease (pooled odds ratio (OR) =1.207, 95% CI: 1.162-1.254), myocardial infarction (OR =1.132, 95% CI: 1.104-1.160), ischemic heart disease (OR =1.129, 95% CI: 1.105-1.154), heart failure (OR =1.050, 95% CI: 1.029-1.072), stroke (OR =1.087, 95% CI: 1.068-1.107), ischemic stroke (OR =1.080, 95% CI: 1.059-1.102), essential hypertension (OR =1.013, 95% CI: 1.010-1.015), coronary atherosclerosis (OR =1.005, 95% CI: 1.004-1.007), and major coronary heart disease event (OR =1.003, 95% CI: 1.002-1.004). Additionally, T2DM was causally related to lower levels of high-density lipoprotein cholesterol (OR =0.965, 95% CI: 0.958-0.973) and apolipoprotein A (OR =0.982, 95% CI: 0.977-0.987) but a higher level of triglycerides (OR =1.060, 95% CI: 1.036-1.084). Moreover, causal effect of glycemic traits on CVDs and lipid traits were also observed. Finally, most results of univariable MR were supported by multivariable MR.

**Conclusion:**

We provided evidence for the causal effects of T2DM and glycemic traits on the risk of CVDs and dyslipidemia. Further investigations to elucidate the underlying mechanisms are warranted.

## Introduction

Evidence from mounting prospective cohort studies has shown that type 2 diabetes mellitus (T2DM) is an independent risk factor of various cardiovascular diseases (CVDs) including coronary heart disease, heart failure (HF), stroke, peripheral artery disease (PAD) and so on (1–3). However, the causal effect of T2DM on CVDs could be confused by body mass index, age, sex, ethnicity, etc. in observational studies. Abnormal glycemic traits in the non-diabetic range, including fasting glucose (FG), fasting insulin (FI), and hemoglobin A1c (HbA_1c_), were reported to be associated with CVDs (2–5). However, there are still conflict findings (6–11). Thus, the association remains uncertain. Patients with T2DM or abnormal glycemic traits were observed to predispose to the development of dyslipidemia such as increased low-density lipoprotein cholesterol (LDL-C), increased triglyceride, and decreased high-density lipoprotein cholesterol (HDL-C) (12). However, whether T2DM or abnormal glycemic trait is a cause or consequence of dyslipidemia is uncertain.

Mendelian randomization (MR) is an approach that relies on genetic variants that are considered to be allocated randomly at birth and is less subject to many confounders than observational studies (13). A previous MR study has investigated the relationship between T2DM and CVDs in a single cohort and revealed causal effects of T2DM on a range of CVDs (14). In our MR study, we pooled the estimates from two independent cohorts to ensure the robustness of the causal effects of T2DM on CVDs. Besides, we took three glycemic traits (FG, FI and HbA_1c_) closely related to T2DM into consideration and conducted multivariable analyses to avoid bias of confounders brought by these traits. We further explored whether the causal effect of T2DM on CVDs was mediated by dyslipidemia using mediation analysis. Additionally, we evaluated whether genetically predicted T2DM or abnormal glycemic traits are causally associated with lipid traits.

## Materials and Methods

### MR and Genome-Wide Association Studies (GWAS) Summary Data

**Figure 1:**
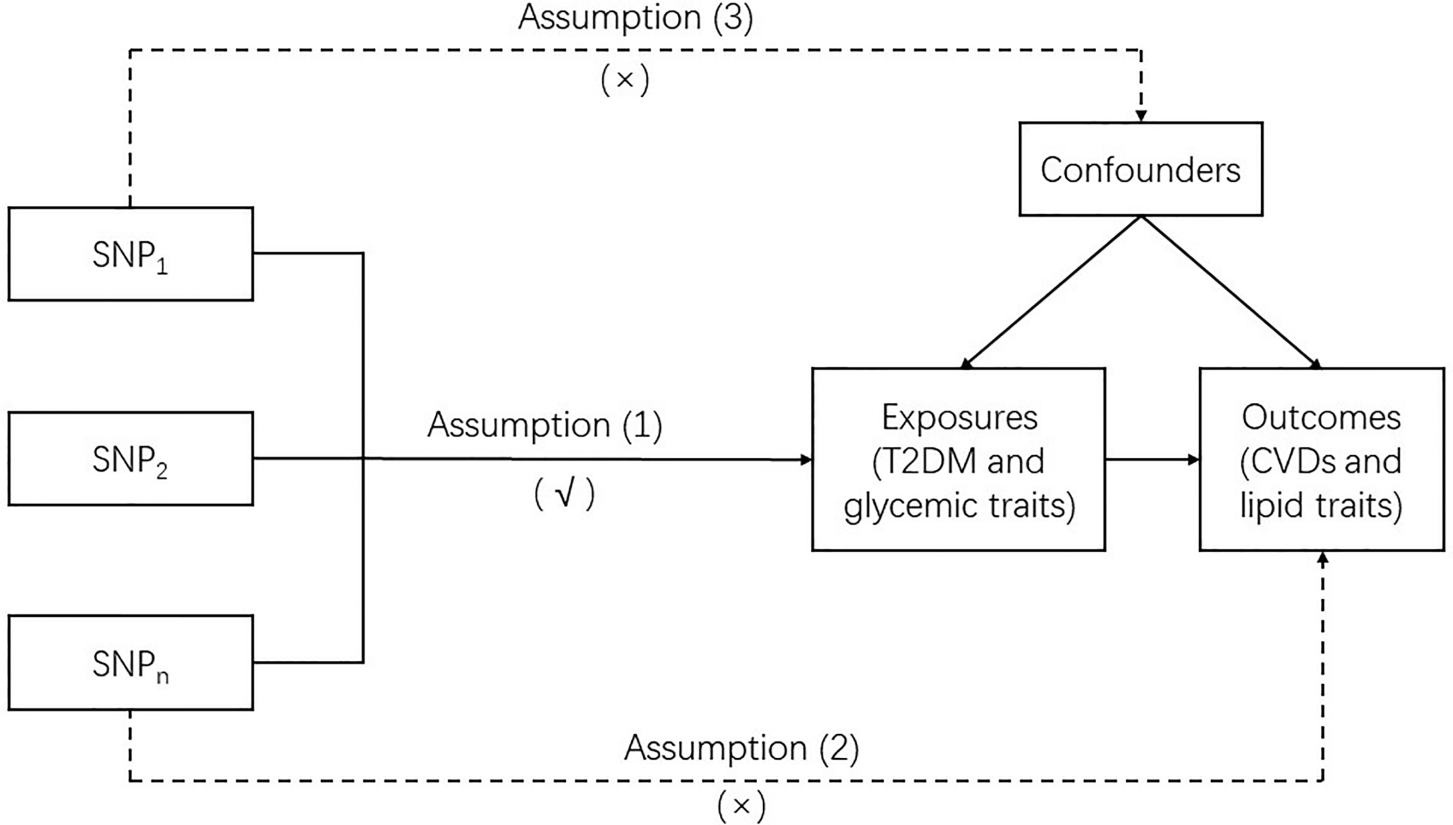
Mendelian randomization model.

MR is a genetic instrumental variable (IV)-based approach that utilizes single nucleotide polymorphisms (SNPs) as IVs to clarify the causal association between exposure and outcome. In this study, two-sample MR was used. Our MR analysis was based on three basic assumptions: (1) the IVs were robustly associated with the exposures (T2DM and glycemic traits); (2) the IVs affected the outcomes (CVDs and lipid traits) merely by their effect on exposures without any other causal pathways, which is also called no pleiotropic effect from the exposures; and (3) the IVs were not associated with any confounders which are present in the relation between the exposures and outcomes. To assure the reliability of the causal link between the exposures and outcomes obtained by MR, none of these assumptions should be violated (**Figure 1**).

The summary-level data were obtained from the OpenGWAS database developed by the MRC Integrative Epidemiology Unit (IEU) (https://gwasmrcieu.ac.uk/). Most of the datasets were publicly available and could be obtained by accessing application programming interfaces through convenient packages in R and Python (15, 16). Details on the phenotypes and consortiums are available in **Supplementary Table 1**.

### IVs for Exposures

We obtained the genetic instruments for T2DM from a meta-analysis of GWASs that included 74124 cases and 824006 controls from the DIAbetes Genetics Replication And Meta-analysis (DIAGRAM) consortium, which was derived from 32 GWASs conducted in populations of European ancestry (17). For the glycemic traits, the IVs for FG and FI were constructed from a meta-analysis of GWASs, which included 52 studies comprising up to approximately 133010 nondiabetic individuals from MAGIC (Meta-Analysis of Glucose and Insulin related traits Consortium) (18). The IVs for HbA_1c_ were obtained from a meta-analysis of 82 cohorts that included up to 88355 European participants (19). All SNPs with a *p* value < 5 × 10^-8^ were considered significant variants associated with phenotypes and included. We excluded SNPs with r^2^ < 0.001 using linkage disequilibrium analysis. To avoid “weak instrument” bias, the F-statistic was calculated according to the formula $F=\frac{R^{2}(n-k-1)}{k(1-R^{2})}$ , where n, k, and R^2^ represent the sample size, the number of SNPs, and the proportion of variance explained by the instrumental variants, respectively (20, 21). An F-statistic value > 10 was regarded as strong enough to avoid weak instrument bias (22). Finally, 286, 35, 18, and 38 SNPs served as IVs for T2DM, FG, FI, and HbA_1c_, respectively (**Supplementary Table 2**).

### GWAS Summary Data for CVDs

A broad spectrum of CVDs were included in our study. Summary statistics were extracted from the Coronary Artery Disease Genome-wide Replication and Meta-analysis (CARDIoGRAM) plus the Coronary Artery Disease (C4D) Genetics (CARDIoGRAMplusC4D) for myocardial infarction (MI) and ischemic heart disease (IHD) (23); from the UKB for coronary atherosclerosis (CA), major coronary heart disease event (MCHDE), essential hypertension (HT), intracerebral hemorrhage and cardiovascular mortality (CM) (24); from the MEGASTROKE Consortium for stroke and ischemic stroke (IS) (25); from the Heart failure Events reduction with Remote Monitoring and eHealth Support (HERMeS) for HF (26); from the BioBank Japan (BBJ) for PAD (27); and from a meta-analysis including 6 studies (The Nord-Trøndelag Health Study (HUNT), deCODE, the Michigan Genomics Initiative (MGI), DiscovEHR, UKB, and the AFGen Consortium) for atrial fibrillation and fluttering (AF) (28).

To ensure the homogeneity of the study population and the reliabilities of the results, each CVD was derived from two independent large-scale cohorts. Therefore, summary-level data of each CVD were also extracted from the FinnGen consortium (study page: https://www.finngen.fi/en/; release 5: https://r5.finngen.fi/). According to the first occurrence, all CVDs were defined by the International Classification of Diseases (ICD)-10. The definition of each CVD is shown in **Supplementary Table 3**.

### GWAS Summary Data for Lipid Traits

We explored the following lipid traits measured in the UKB (20): HDL-C, LDL-C, triglycerides, apolipoprotein A (apoA), apolipoprotein B (apoB), and lipoprotein(a) [Lp(a)]. In addition, HDL-C, LDL-C, and triglycerides were explored again, utilizing the data from the Global Lipids Genetics Consortium (GLGC) to strengthen the credibility of the causal effects (29). We failed to reconduct analyses for the remaining three lipid traits due to the lack of data.

### Statistical Analyses

For the primary analyses, the univariable inverse variance-weighted (IVW) method was used to investigate the effects of different exposures on outcomes (30). Using the Wald ratio estimates of each SNP, the IVW method combines them into one cumulative causal estimate. Since the results of the IVW method could be affected by undetectable invalid IV bias or potentially unbalanced pleiotropy, different sensitivity analyses were performed to detect the robustness and validity of the MR results. First, the MR–Egger method was used to confirm the consistency of MR results and explore the horizontal pleiotropy effect through the intercept (31). Second, the heterogeneity of IVW and MR–Egger was calculated (32). A fixed-effects model was adopted to assess the IVW estimates when there was no significant heterogeneity; otherwise, a random-effects model was used. Third, we applied the MR Pleiotropy Residual Sum and Outlier (MR-PRESSO) method to recognize outlying SNPs, which might cause horizontal pleiotropy effects, and examine whether the causal effect would change after removing these outliers (33). Fourth, the weighted median, simple mode, and weighted mode were also employed to test the potential horizontal pleiotropy (34). Except for the analyses of apoA, apoB, and Lp(a), estimates of the causal effect from two independent cohorts were pooled using fixed-effects meta-analysis. It is also important to further evaluate whether the risk of CVDs in T2DM was mediated by dyslipidemia. Therefore, two-step MR was conducted to calculate the mediation effects of lipid traits in the relationship between T2DM and risk of CVDs (35).

For the complementary analyses, multivariable MR (MVMR) analysis was conducted using the IVW method, which incorporates different phenotypes as a single exposure into the MR analysis. In this study, since the relationship between T2DM and three glycemic traits was considered, we fitted a model with T2DM, FG, FI, and HbA_1c_ to detect which phenotypes appeared to be significantly associated with the risk of CVDs or abnormal lipid traits.

All MR analyses were performed using R (version 4.1.1). In the univariable MR step, estimates were obtained with the “TwoSampleMR” package, recognizing outliers with the “MR-PRESSO” package. The MVMR was conducted with the “MendelianRandomization” package. MR results were reported as odd ratios (ORs) with 95% confidence intervals (CIs) per standard deviation or odds of objectively measured continuous or dichotomous variables. For the primary analyses, since we included analyses of 18 outcomes, a Bonferroni-corrected *p* value less than 0.05 divided by 18 (that is, 0.0028) was regarded as a significant causal association to adjust for multiple testing. A *p* value between 0.05 and 0.0028 was considered suggestive of a potential association.

## Results

### Primary Analyses

**Figure 2:**
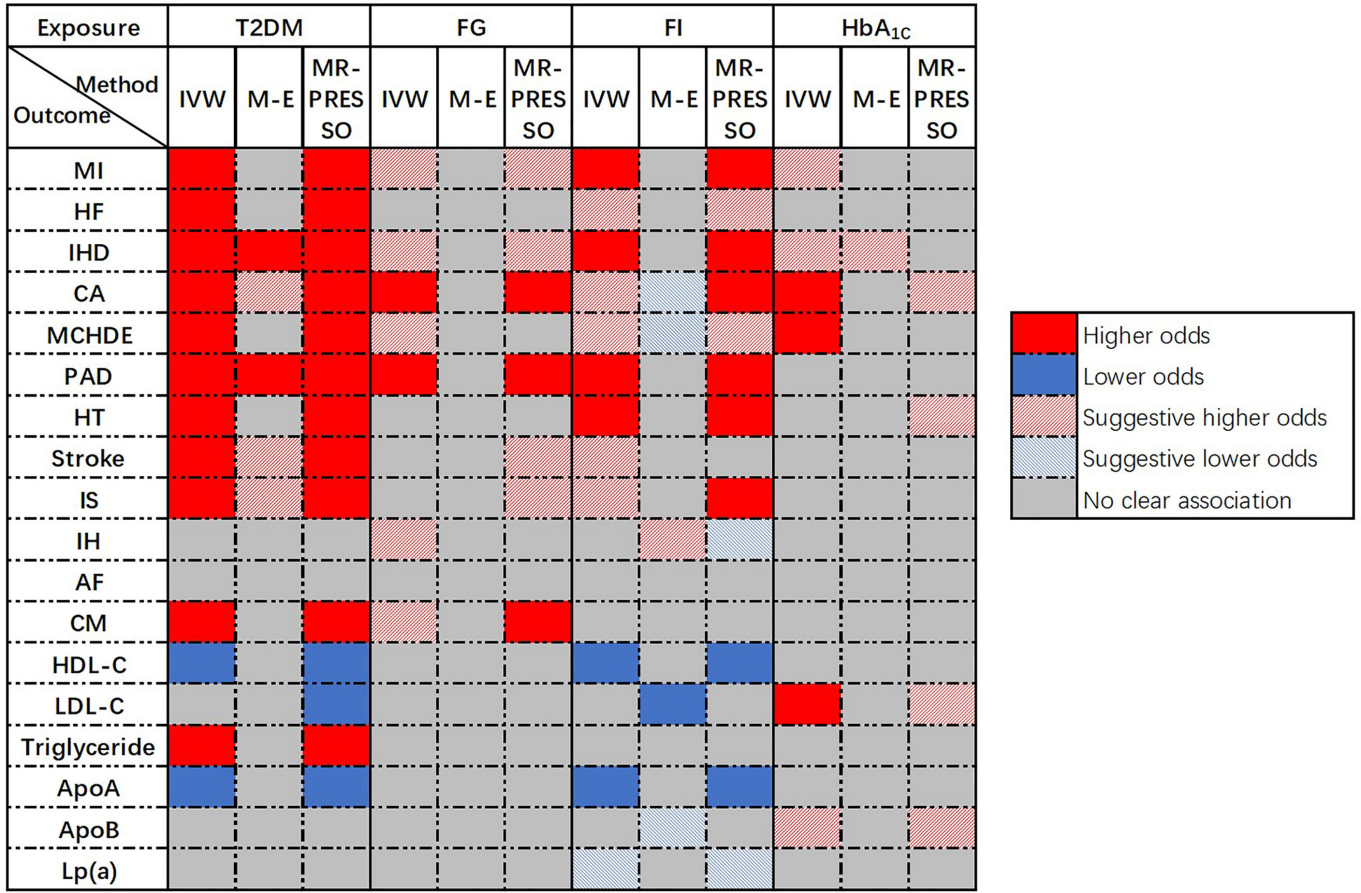
Overview of the main results of Univariable MR.

Univariable MR was conducted and 286, 35, 18, and 38 SNPs associated with T2DM, FG, FI, and HbA_1c_, respectively, were selected as IVs. A flow chart of the study was presented in **Supplementary Figure 1**. An overview of the main results of the primary analyses was shown in **Figure 2**.

#### Causal Association of T2DM With CVDs and Lipid Traits

**Figure 3:**
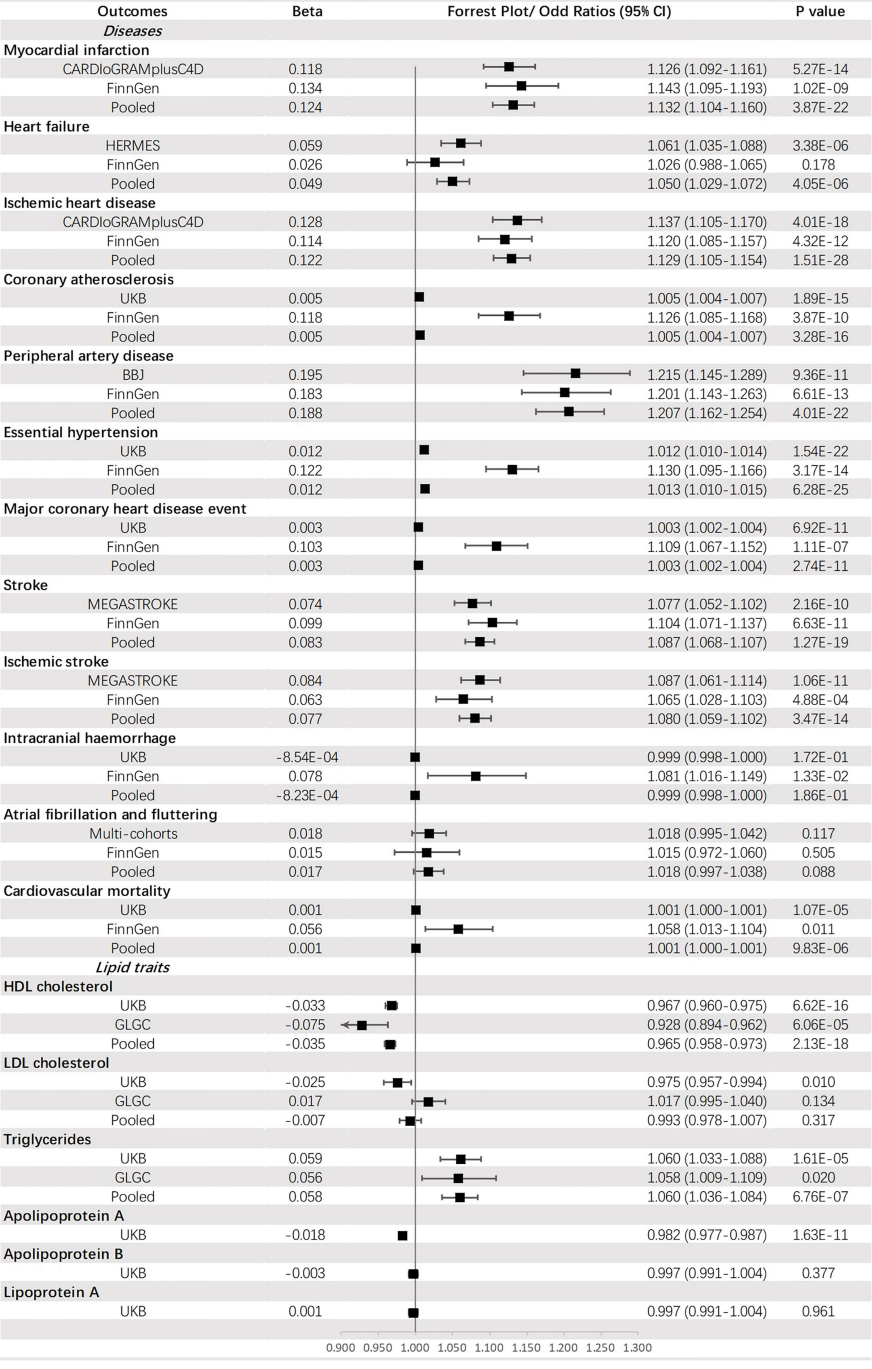
The association between type 2 diabetes mellitus and outcomes.

Genetically predicted T2DM was significantly associated with (ordered from largest estimate decreasing): PAD (OR = 1.207, 95% CI: 1.162-1.254, *p* = 4.01 × 10^-22^), MI (OR=1.132, 95% CI: 1.104-1.160, *p* = 3.87 × 10^-22^), IHD (OR = 1.129, 95% CI: 1.105-1.154, *p* = 1.51 × 10^-28^), stroke (OR = 1.087, 95% CI: 1.068-1.107, *p* = 1.27 × 10^-19^), IS (OR = 1.080, 95% CI: 1.059-1.102, *p* = 1.40 × 10^-3^), HF (OR = 1.050, 95% CI: 1.029-1.072, *p* = 4.05 × 10^-6^), HT (OR = 1.013, 95% CI: 1.010-1.015, *p* = 6.28 × 10^-25^), CA (OR = 1.005, 95% CI: 1.004-1.007, *p* = 3.28 × 10^-16^), MCHDE (OR = 1.003, 95% CI: 1.002-1.004, *p* = 2.74 × 10^-11^), and CM (OR = 1.001, 95% CI: 1.000-1.001, *p* = 9.83 × 10^-6^). We also found T2DM was causally related to lower levels of HDL-C (OR = 0.965, 95% CI: 0.958-0.973, *p* = 2.13 × 10^-18^) and apoA (OR = 0.982, 95% CI: 0.977-0.987, *p* = 1.63 × 10^-11^) but a higher level of triglycerides (OR = 1.060, 95% CI: 1.036-1.084, *p* = 6.76 × 10^-7^) (**Figure 3**).

#### Causal Association of Glycemic Traits With CVDs and Lipid Traits

**Figure 4:**
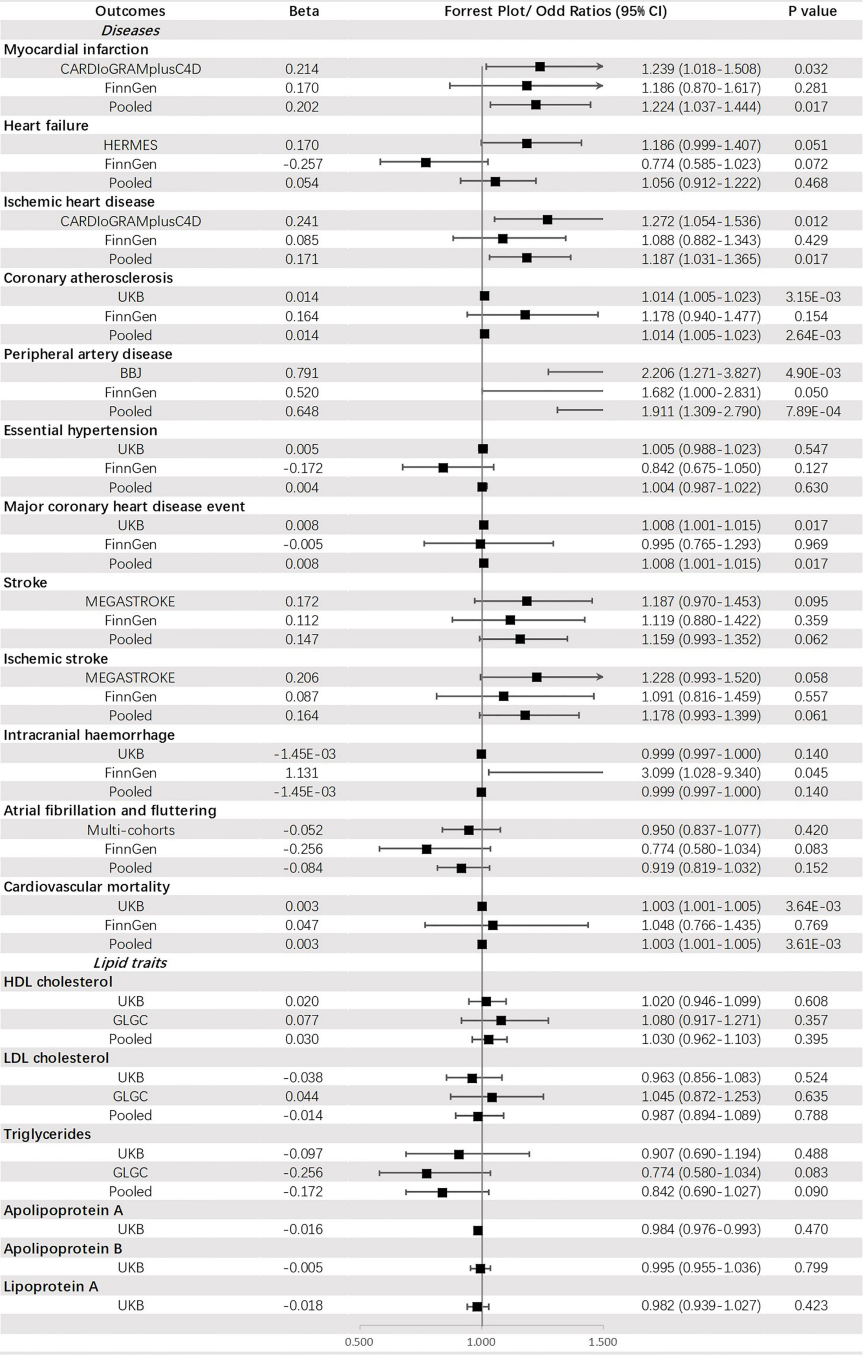
The association between fasting glucose (mmol/mol) and outcomes.

Genetically predicted FG was significantly associated with PAD (OR = 1.911, 95% CI: 1.309-2.790, *p* = 7.89 × 10^-4^) and CA (OR = 1.014, 95% CI: 1.005-1.023, *p* = 2.64 × 10^-3^). Additionally, a potential increased risk was observed for IHD (OR =1.187, 95% CI: 1.031-1.365, *p* value = 0.017), MCHDE (OR =1.008, 95% CI: 1.001-1.015, *p* value = 0.017), and CM (OR = 1.003, 95% CI: 1.001-1.005, *p* = 3.61 × 10^-3^) (**Figure 4**).

**Figure 5:**
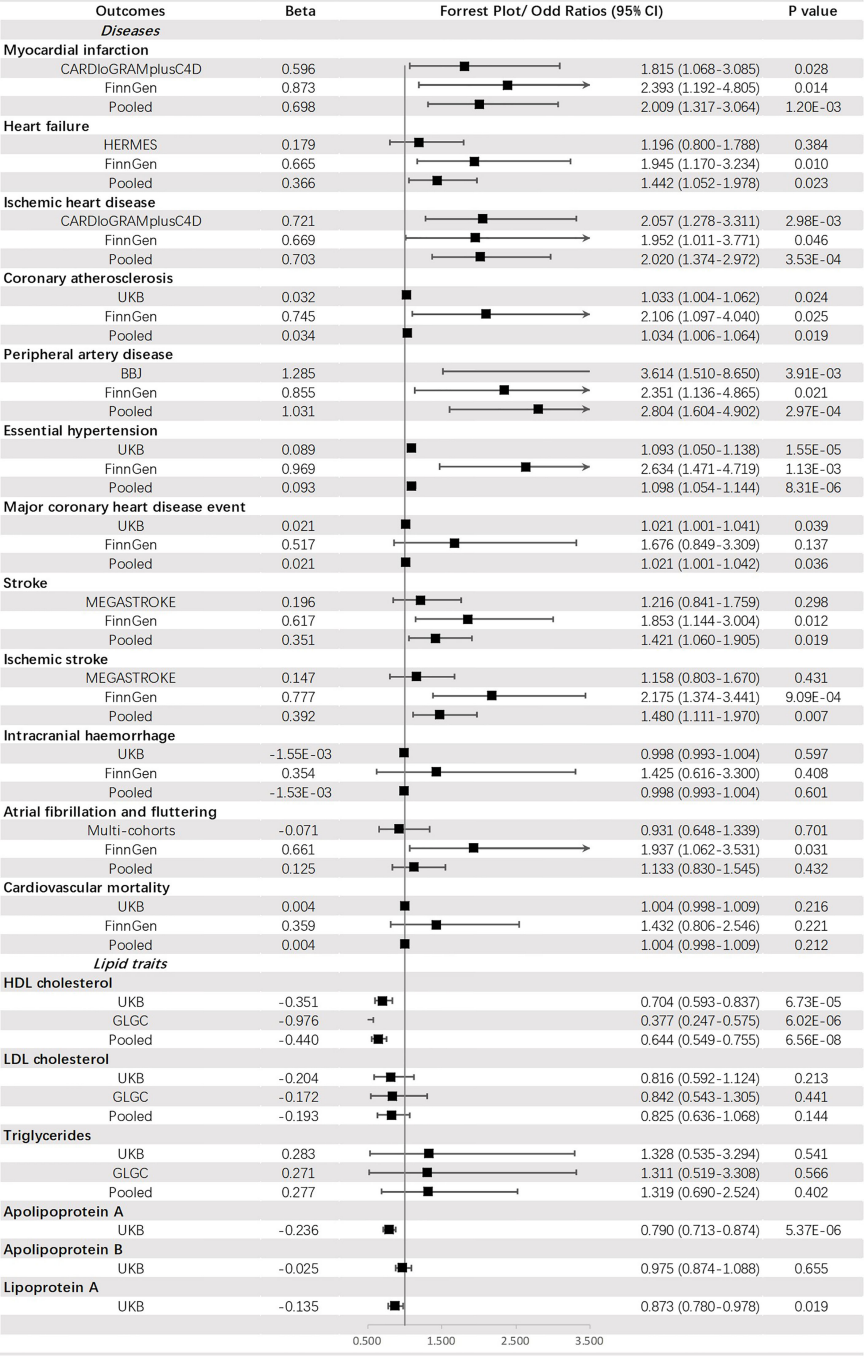
The association between fasting insulin (mmol/mol) and outcomes.

Genetically predicted FI was suggested to be positively corelated with PAD (OR = 2.804, 95% CI: 1.604-4.902, p = 2.97 × 10_-4_), IHD (OR = 2.020, 95% CI: 1.374-2.972, p = 3.53 × 10_-4_), MI (OR = 2.009, 95% CI: 1.317-3.064, *p* = 1.20 × 10^-3^) and HT (OR = 1.098, 95% CI: 1.054-1.144, *p* = 8.31 × 10^-6^) but negatively associated with HDL-C (OR = 0.644, 95% CI: 0.549-0.755, *p* = 6.56 × 10^-8^) and apoA (OR = 0.790, 95% CI: 0.713-0.874, *p* = 5.37 × 10^-6^). An indistinct relation to HF (OR = 1.442, 95% CI: 1.052-1.978, *p* = 0.023), stroke (OR = 1.421, 95% CI: 1.060-1.905, *p* = 0.019), IS (OR =1.480, 95% CI: 1.111-1.970, *p* = 0.007), CA (OR = 1.034, 95% CI: 1.006-1.064, *p* = 0.019), MCHDE (OR = 1.021, 95% CI: 1.001-1.042, *p* = 0.036) and lower level of Lp(a) (OR = 0.873, 95% CI: 0.780-0.978, *p* = 0.019) was also found (**Figure 5**).

**Figure 6:**
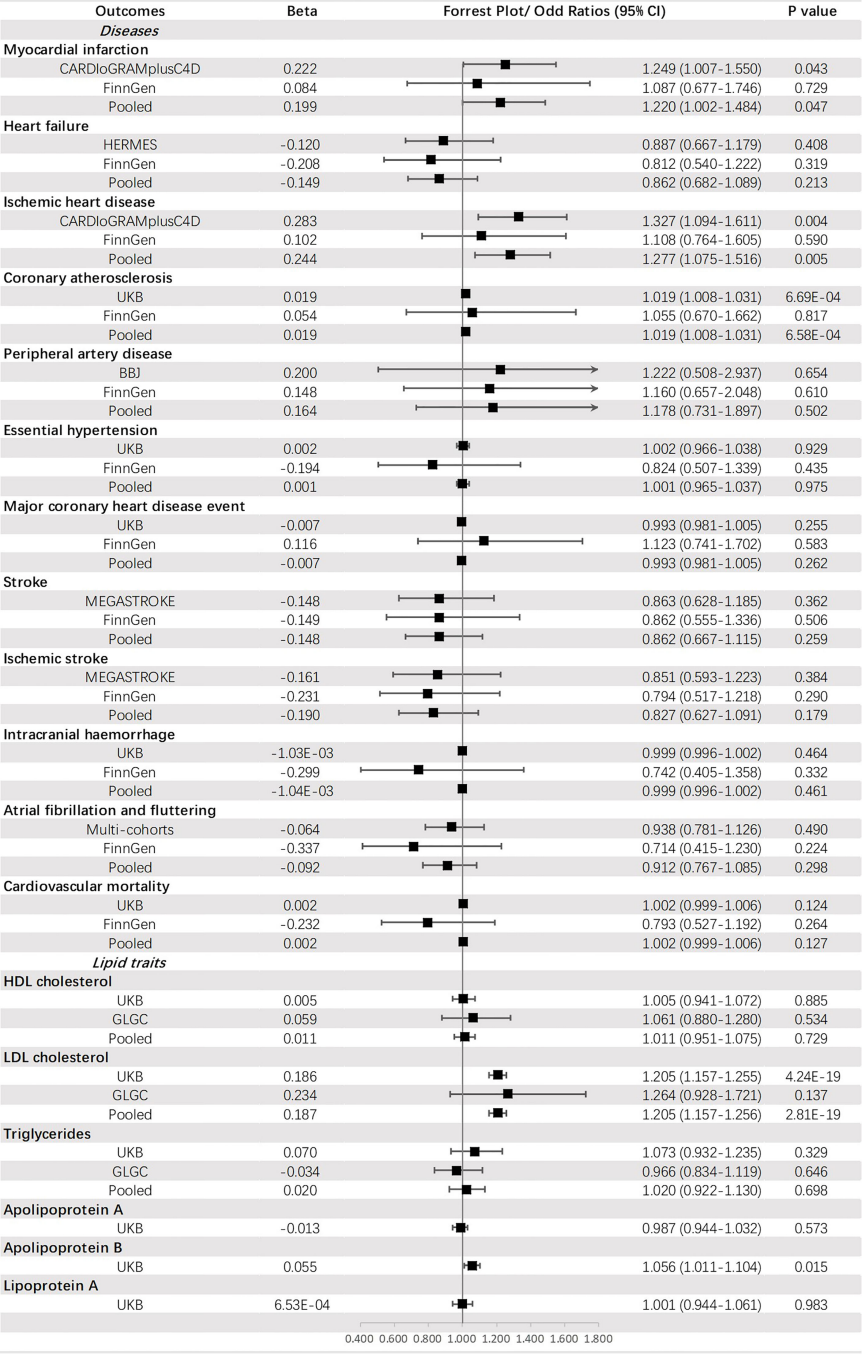
The association between HbA_1c_ (mmol/mol) and outcomes.

Genetically predicted HbA_1c_ was significantly associated with CA (OR = 1.019, 95% CI: 1.008-1.031, *p* = 6.58 × 10^-4^) and an increased level of LDL-C (OR =1.205, 95% CI: 1.157-1.256, *p* = 2.81 × 10^-19^). A suggestive causal effect was also observed for IHD (OR =1.277, 95% CI: 1.075-1.516, *p* = 0.005) and MI (OR =1.220, 95% CI: 1.002-1.484, *p* = 0.047), and increased level of apoB (OR =1.056, 95% CI: 1.011-1.104, *p* = 0.015) (**Figure 6**).

#### Robustness of the Primary Analyses

In the univariable MR analysis, we observed significant heterogeneities in some estimates. We adopted a random-effects model to adjust the IVW estimates, as mentioned in the Methods section. The MR–Egger intercepts were mostly insignificantly larger or less than zero, eliminating part of the horizontal pleiotropy. Using the MR-PRESSO method, several outliers were identified during the analysis, and in most cases, the results remained consistent with the original ones after removing these outliers. In addition, estimates using MR–Egger, weighted median, simple mode, and weighted mode were also calculated, and the results suggested relatively high robustness (**Supplementary Tables 4–7**).

#### Mediation Analyses

We performed mediation analyses using two-step MR to clarify whether the causal effect of T2DM on the risk of CVDs was mediated by dyslipidemia. HDL-C, triglycerides and apoA were chosen as potential mediators since they showed a significant association with T2DM in the primary analyses. We found HDL-C explained a small part of the casual effects of T2DM on the risk of MI, CA, PAD, and HT, and the mediation proportions were 7.4%, 12.8%, 10.6%, and 5.9%, respectively (**Supplementary Table 8**). Triglycerides and apoA were also mediators of the causal association between T2DM and several types of CVDs (**Supplementary Tables 9, 10**).

### Complementary Analyses

MVMR was conducted for outcomes with significant estimates in primary analyses. Most results of univariable MR were supported by MVMR. However, the causal effects of T2DM on HF, FI on IHD, FG on PAD, FG on CA, and HbA_1c_ on CA were not found following adjustment for the other three exposures. An inverse association between T2DM and level of LDL-C was observed using multivariable analysis. Detailed results of MVMR were presented in **Supplementary Table 11**.

## Discussion

In this study, a two-sample MR method utilizing GWAS summary-level data was applied to explore the causal association of T2DM and glycemic traits (FG, FI, and HbA_1c_) with a wide range of CVDs as well as lipid traits [HDL-C, LDL-C, triglycerides, apoA, apoB, and Lp(a)]. The primary analyses found evidence that genetically predicted T2DM was associated with various types of CVDs including MI, HF, IHD, CA, MCHDE, PAD, HT, stroke, IS, and CM. Additionally, T2DM was associated with a higher level of triglycerides but lower levels of HDL-C and apoA. Moreover, causal effect of glycemic traits on CVDs and lipid traits were also observed. FI was associated with higher levels of HDL-C and triglycerides, and HbA_1c_ was associated with a higher level of LDL-C. Sensitivity analyses suggested the robustness of the causal effects. As a complementary analysis, MVMR was conducted which incorporated the four exposures into a model. Most results of univariable MR were supported by multivariable MR.

### T2DM and CVDs

Our findings are in line with the previous MR studies supporting casual effects of T2DM on various CVDs (14). We here provide evidence supporting additional effects of T2DM on HT, CA, and CM. However, in our MR study, the causal association between T2DM and HF disappeared after adjusting for multiple variables, which was inconsistent with the results of Liu et al. Moreover, multiple epidemiological studies had consistent results with ours (36–38). However, Wei et al. investigated the association between T2DM and several CVDs using phenotype and genetic predisposition data from the China Kadoorie Biobank. At the observational level, a significantly positive correlation was observed for all CVD outcomes but not for major coronary events, cardiovascular mortality, or total stroke at the genetic level (39). This discrepancy between observational and genetic results suggests that the causal link between T2DM and CVDs remains largely to be determined. Fortunately, we found the causal effect of T2DM on these diseases.

Unfortunately, we failed to obtain a causal effect of T2DM on AF and IH. No association of T2DM with AF was also found by Hadi et al. using the MR approach (40). However, the Framingham Heart Study observed a 1.4- to 1.6-fold greater risk of AF in diabetic individuals after adjusting for age and other risk factors (41). One hypothesis about this inconsistency was that hypertension and obesity are the common comorbidities of T2DM, which could result in confounder bias in the observational studies, but not in the MR studies (40). In another MR study focusing on T2DM and cerebral disease, the researcher also found the null association between T2DM and IH even subdividing IH into lobar IH and deep IH (42).

### Glycemic Traits and CVDs

Previous MR results showed that HbA_1c_ has a causal role in coronary artery disease but FG does not (43). However, in our study, FG was also shown to have a causal effect on several types of coronary artery diseases. Notably, with regard to glycemic traits, some epidemiological evidence did not support our findings of their causal effects on CVDs. The results derived from the Jackson Heart Study (JHS) revealed that dysglycemia, including higher levels of FG and HbA_1c_, was associated with an increased risk of HF (44), which failed to reappear in our MR study. We inferred that ethnic variation may have led to the difference in results since the JHS recruited mainly Black participants from Mississippi. Justin et al. found that even stratifying the HF into HF with preserved ejection fraction and reduced ejection fraction, the causal chain was still there (45).

### T2DM-Related Traits and Lipid Traits

As suggested by our data, T2DM negatively affected HDL-C. Accordingly, a causal effect of T2DM on the decreased level of apoA (a main component of HDL-C) was also observed. Decreased HDL-C levels in T2DM patients was observed in a previous observational study (46). One explanation was that insulin resistance in T2DM patients might be responsible for the low level of HDL-C (47). As far as we know, our study is the first to provide evidence on the causal association between T2DM and a lower level of HDL-C from the genetic level. In our study, T2DM was also found to be causally associated with triglycerides, which is consistent with a previous MR study (48).

In our study, HbA_1c_ was causally associated with an increased level of LDL-C. A transversal observational study found that oxidized LDL-C, rather than total LDL-C, was associated with HbA_1c_ in the non-diabetic range (49). However, we could not obtain data to stratify LDL-C into subgroups to explore this relation further. In our study, FI was found to negatively affected on Lp(a), and it showed a potentially negative effect. Conversely, Buchmann et al. found no evidence of a causal effect of FI on Lp(a) using rs780094 and rs10195252 (SNPs associated with FI) as IVs through the MR method (50). The possible mechanism by which insulin modulates Lp(a) synthesis may be that increased insulin levels promote the progression of insulin resistance and, under these circumstances, reduce the synthesis of Lp(a) (51).

This study had several strengths. As we known, our study is the first to demonstrate a causal association of T2DM and the related glycemic traits with a broad spectrum of CVDs and dyslipidaemia using MR and employing large GWASs data. Two-sample MR method was utilized, which eliminated residual confounding as much as possible. For the outcomes of CVDs, we utilized two highly representative and independent cohorts to stabilize the results of our causal inference. Moreover, the F-statistics of the genetic variants were mostly more than 10, which indicated that the genetic variants were strong enough to be IVs for exposure. Since T2DM and glycemic traits may interact mutually from the pathogenesis of diseases, multivariable MR was applied to adjust the estimate for these exposures. Ultimately, in order to evaluate the robustness of MR results, different MR methods and tests of heterogeneity and pleiotropy were conducted as additional means of sensitivity analyses.

Some limitations could not be ignored. First, most participants included in the GWASs were of European ancestry. Consequently, whether our findings are generalizable to other populations and regions remains to be determined. Second, it is scarcely possible to remove all pleiotropy in MR studies, and some undetected pathways may play a role as confounders between exposures and outcomes, biasing our results. Third, we could obtain only summary-level GWAS data, failing to conduct further investigation on the sex-, age-, and specific type of exposure-related effect on the outcomes. Moreover, the results of MVMR were possibly biased by overfitting from the multivariable model and attenuated or amplified the estimates of effect, which was also observed in this study as compared to the univariable MR. Last, since the glycemic and lipid traits were predisposed as continuous variables, we assumed that the relationship between T2DM or CVDs was linear, which could be inconsistent with the actual situation.

In conclusion, our MR study provides further evidence that T2DM and its related glycemic traits play a causal role in the increased risk of various CVDs and dyslipidemia. The findings should be interpreted to strengthen the awareness of early detection of T2DM and its related glycemic traits. Further work using individual-level data or basic science approaches to investigate the mechanisms mediating these causal associations is warranted.

## Data Availability Statement

The original contributions presented in the study are included in the article/**Supplementary Material**. Further inquiries can be directed to the corresponding authors.

## Author Contributions

MH and LL designed the study. MH, LZ, and LL undertook the data analyses with feedback from TT and XC. MH and LL co-wrote the paper. TT and XC reviewed and provided important suggestions for the manuscript. TT and XC are the guarantors of this study. All authors contributed to the article and approved the submitted version.

## Funding

This work was supported by the Grants from the National Natural Science Foundation of China (81720108005 and 82030016 to XC; 81974037 and 82170394 to TT), the Foundation for Innovative Research Groups of National Natural Science Foundation of China (82021005 to XC), and the 2017 Chang Jiang Scholars Program (T2017073 to XC).

## Conflict of Interest

The authors declare that the research was conducted in the absence of any commercial or financial relationships that could be construed as a potential conflict of interest.

## Publisher’s Note

All claims expressed in this article are solely those of the authors and do not necessarily represent those of their affiliated organizations, or those of the publisher, the editors and the reviewers. Any product that may be evaluated in this article, or claim that may be made by its manufacturer, is not guaranteed or endorsed by the publisher.

## Abbreviations

T2DM, type 2 diabetes mellitus; CVD, cardiovascular disease; FG, fasting glucose; FI, fasting insulin; HbA_1c_, hemoglobin A1c; MR, Mendelian randomization; GWAS, genome-wide association study; IV, instrumental variable; SNP, single nucleotide polymorphism; MI, myocardial infarction; IHD, ischemic heart disease; CA, coronary atherosclerosis; MCHDE, major coronary heart disease event; HT, essential hypertension; CM, cardiovascular mortality; IS, ischemic stroke; HF, heart failure; PAD, peripheral artery disease; AF, atrial fibrillation and fluttering; HDL-C, high-density lipoprotein cholesterol; LDL-C, low-density lipoprotein cholesterol; ApoA, apolipoprotein A; ApoB, apolipoprotein B; Lp(a), lipoprotein(a); IVW, inverse variance-weighted; MVMR, multivariable MR.
