# Supplementary material for: Causal Association of Type 2 Diabetes Mellitus and Glycemic Traits With Cardiovascular Diseases and Lipid Traits: A Mendelian Randomization Study
Source: Front Endocrinol (Lausanne). 2022 Apr 22;13:840579. doi: 10.3389/fendo.2022.840579 (PMC9072667; doi:10.3389/fendo.2022.840579)
Supplement: Supplementary file 1 [file DataSheet_1.pdf]

Causal Association of Type 2 Diabetes Mellitus and Glycemic Traits with Cardiovascular Events and Lipid Traits: A Mendelian Randomization Study

Supplementary Material

Mingkai Huang[1,2†], Loum-Davadi Laina-Nicaise[1,2†], Lingfeng Zha[1,2†], Tingting Tang[1,2*], Xiang Cheng[1,2*]

[1]Department of Cardiology, Union Hospital, Tongji Medical College, Huazhong University of Science and Technology, Wuhan, China

[2]Key Laboratory of Biological Targeted Therapy of the Ministry of Education, Union Hospital, Tongji Medical College, Huazhong University of Science and Technology, Wuhan, China

Figure S1 Study overview.

Table S1 Phenotype source and description.

Table S2 Genetic variants associated with exposures.

Table S3 Definition and sources for CVDs in FinnGen.

Table S4 The causal effect of genetically predicted type 2 diabetes mellitus on outcomes in different studies.

Table S5 The causal effect of genetically predicted FG on outcomes in different studies.

Table S6 The causal effect of genetically predicted FI on outcomes in different studies.

Table S7 The causal effect of genetically predicted HbA_{1c} on outcomes in different studies.

Table S8 HDL cholesterol as a mediator in the causal pathway between type 2 diabetes mellitus and risk of CVDs.

Table S9 Triglycerides as a mediator in the causal pathway between type 2 diabetes mellitus and risk of CVDs.

Table S10 Apolipoprotein A as a mediator in the causal pathway between type 2 diabetes mellitus and risk of CVDs.

Table S11 Multivariable Mendelian randomization using IVW method.

Supplementary Figure

Figure S1 Study Overview.

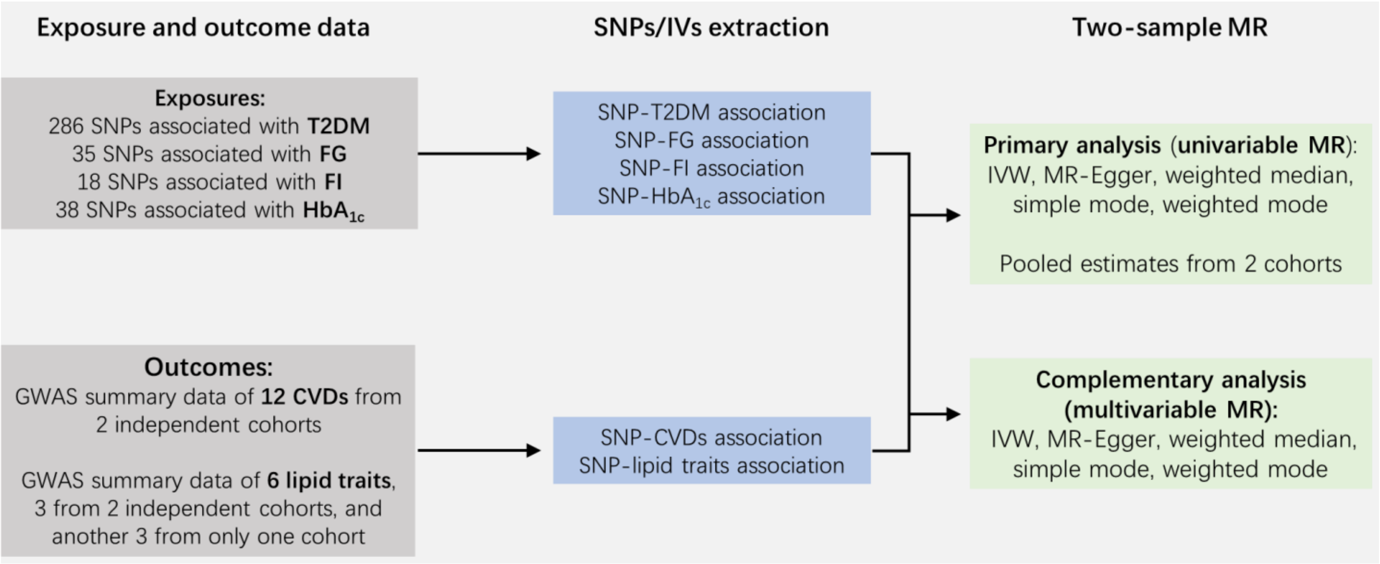

Supplementary Tables

Table S1 Phenotype source and description.

Phenotypes	Consortium or study	No. of cases	No. of controls	Data type	GWAS ID
**Exposure**					
**Type 2 diabetes mellitus**	DIAGRAM	74124	824006	Binary	NA
**Fasting blood glucose**	MAGIC	NA	NA	Continuous	NA
**Fasting insulin**	MAGIC	NA	NA	Continuous	NA
**HbA_{1c}**	MAGIC	NA	NA	Continuous	NA
**CVDs**					
**Myocardial infarction**	CARDIoGRAMplusC4D / FinnGen	43676/4065	128199/ 85760	Binary	ieu-a-798/ finn-a-I9_MI
**Heart failure**	HERMES/ FinnGen	47309/8016	930014/ 75137	Binary	ebi-a-GCST009541/ finn-a-HEARTFAIL

**Ischemic heart disease**	CARDIoGRAMplusC4D / FinnGen	60801/ 10739	123504/ 85760	Binary	ieu-a-7/ finn-a-I9_ISCHHEART
**Coronary atherosclerosis**	UKB/ FinnGen	14334/7661	34860/ 85760	Binary	ukb-d-I9_CORATHER / finn-a-I9_CORATHER
**Major coronary heart disease event**	UKB/ FinnGen	10157/7123	351037/ 89376	Binary	ukb-d-I9_CHD/ finn-a-I9_CHD
**Peripheral artery disease**	BBJ/ FinnGen	7114/2398	475964/ 92349	Binary	bbj-a-144/ finn-a-I9_PAD
**Essential hypertension**	UKB/ FinnGen	54358/ 22142	408652/ 69160	Binary	ukb-b-12493/ finn-a-I9_HYPTENS
**Stroke**	MEGASTROKE/ FinnGen	40585/7144	406111/ 75420	Binary	ebi-a-GCST006906/ finn-a-C_STROKE
**Ischemic stroke**	MEGASTROKE/ FinnGen	34217/4026	406111/ 90211	Binary	ebi-a-GCST006908/ finn-a-I9_STR_EXH
**Intracerebral hemorrhage**	UKB/ FinnGen	1253/1149	359941/ 90757	Binary	ukb-d-I9_INTRACRA/ finn-a-I9_ICH
**Atrial fibrillation and fluttering**	Multi-cohorts[a]/ FinnGen	62620/7244	970216/ 56378	Binary	ebi-a-GCST006414/ finn-a-I9_AF
**Cardiovascular mortality**	UKB/ FinnGen	1597/3480	359597/ 93019	Binary	ukb-d-I9_K_CARDIA/ finn-a-I9_K_CARDIAC
**Lipid traits**					
**HDL cholesterol**	UKB/ GLGC	NA	NA	Continuous	ukb-d-30760_irnt/ ieu-a-299
**LDL cholesterol**	UKB/ GLGC	NA	NA	Continuous	ukb-d-30780_irnt/ ieu-a-300
**Triglycerides**	UKB/ GLGC	NA	NA	Continuous	ukb-d-30870_irnt/

					ieu-a-302
**Apolipoprotein A**	UKB	NA	NA	Continuous	ukb-d-30630_irnt
**Apolipoprotein B**	UKB	NA	NA	Continuous	ukb-d-30640_irnt
**Lipoprotein A**	UKB	NA	NA	Continuous	ukb-d-30790_irnt

[a]From a meta-analysis of six contributing studies (The Nord-Trøndelag Health Study (HUNT), deCODE, the Michigan Genomics Initiative (MGI), DiscovEHR, UK Biobank, and the AFGen Consortium)

Table S2 Genetic variants associated with exposures.

SNP	Chromosome	Gene	Beta	SE	*p* value	F-statistics
**Type 2 diabetes mellitus (286)**						285.95
**rs1005752**	15	HMG20A	0.077	0.007	2.50E-29	323.76
**rs10096633**	8	LPL	0.068	0.010	1.10E-12	92.56
**rs10097617**	8	TP53INP1	0.039	0.007	3.30E-11	102.93
**rs10193538**	2	BNIPL	0.039	0.007	8.90E-09	97.99
**rs10195252**	2	GRB14/COBLL1	0.068	0.005	6.00E-25	424.22
**rs10228066**	7	DGKB	0.068	0.007	1.10E-28	311.11
**rs10406431**	19	GIPR	0.049	0.005	9.60E-14	224.32
**rs1042725**	12	HMGA2	0.049	0.007	1.80E-13	162.49
**rs1061810**	11	HSD17B12	0.049	0.007	6.00E-13	133.88
**rs10750397**	11	ETS1	0.049	0.007	8.30E-13	131.08
**rs10757283**	9	CDKN2A/B	0.104	0.009	1.70E-41	558.65
**rs10811660**	9	CDKN2A/B	0.239	0.010	1.40E-115	1530.24
**rs10830963**	11	MTNR1B	0.095	0.007	4.80E-43	492.92
**rs10842994**	12	KLHDC5	0.077	0.007	4.10E-20	247.14
**rs10882101**	10	HHEX/IDE	0.058	0.010	1.40E-08	154.26
**rs10893829**	11	ETS1	0.058	0.010	1.30E-10	81.30
**rs10937721**	4	WFS1	0.058	0.010	1.50E-08	154.26
**rs10938398**	4	GNPDA2	0.049	0.007	3.60E-12	159.37
**rs10954772**	8	PURG	0.039	0.007	1.80E-09	88.10
**rs10962**	17	HNF1B	0.049	0.010	9.90E-10	80.60
**rs10974438**	9	GLIS3	0.049	0.007	1.50E-14	149.81

**rs11042596**	11	INS/IGF2	0.039	0.005	2.00E-08	127.50
**rs11063028**	12	CCND2	0.058	0.007	8.50E-11	134.46
**rs11070332**	15	LTK	0.049	0.005	1.10E-13	209.75
**rs11137820**	9	MTND2P8	0.039	0.007	2.90E-08	100.34
**rs11202627**	10	PTEN	0.058	0.010	4.70E-08	81.30
**rs11257655**	10	CDC123/CAMK1D	0.086	0.007	1.50E-32	343.78
**rs1127215**	1	PTGFRN	0.049	0.005	1.60E-13	221.77
**rs113414093**	2	CRYBA2	0.113	0.020	6.60E-09	57.48
**rs11496066**	7	FBXL13	0.077	0.014	1.10E-08	118.49
**rs115505614**	5	PAM	0.174	0.015	1.30E-30	181.76
**rs11642430**	16	FAM57B	0.039	0.005	2.20E-09	138.40
**rs11680058**	2	FAM49A	0.058	0.010	1.40E-08	76.77
**rs11688682**	2	GLI2	0.049	0.007	4.20E-09	128.16
**rs11699802**	20	CEBPB	0.039	0.007	1.80E-11	102.31
**rs117001013**	22	YWHAH	0.068	0.012	1.70E-08	59.82
**rs11708067**	3	ADCY5	0.086	0.007	5.20E-32	354.80
**rs11709077**	3	PPARG	0.131	0.011	1.80E-36	312.37
**rs117483894**	15	TCF12	0.095	0.016	3.90E-08	41.06
**rs11759026**	6	CENPW	0.068	0.007	2.40E-18	221.79
**rs11820019**	11	CCND1	0.148	0.020	5.10E-12	60.41
**rs11842871**	13	HMGB1	0.039	0.007	1.20E-08	79.25
**rs11926707**	3	KIF9	0.239	0.042	2.10E-08	601.28
**rs12001437**	9	UBAP2	0.039	0.007	2.80E-10	96.01
**rs12140153**	1	PATJ	0.068	0.012	1.30E-08	59.82
**rs12454712**	18	BCL2A	0.049	0.005	4.60E-13	216.58
**rs1260326**	2	GCKR	0.068	0.005	6.50E-25	417.20
**rs12640250**	4	LCORL	0.039	0.005	3.70E-08	116.25
**rs12642790**	4	SCD5	0.039	0.007	4.40E-10	92.43
**rs12719778**	8	BOP1	0.039	0.005	5.00E-09	143.24
**rs12811407**	12	FBRSL1	0.049	0.007	1.70E-12	143.76
**rs12910825**	15	PRC1	0.049	0.007	1.60E-15	149.81
**rs12920022**	16	SPG7	0.049	0.007	3.40E-09	87.38
**rs1296328**	4	PABPC4L	0.039	0.007	3.50E-08	101.94
**rs13024606**	2	GRB14/COBLL1	0.086	0.016	1.70E-08	41.62
**rs13041756**	20	NKX2.2	0.058	0.010	1.40E-08	62.43
**rs13065698**	3	MBNL1	0.049	0.007	8.10E-13	156.06
**rs13085136**	3	SHQ1	0.077	0.012	1.50E-08	60.97
**rs1316776**	5	DMGDH	0.049	0.007	2.60E-12	147.93
**rs13262861**	8	ANK1	0.068	0.010	4.00E-12	123.68

**rs13426680**	2	CYTIP	0.086	0.012	6.70E-10	65.89
**rs1359790**	13	SPRY2	0.086	0.007	2.40E-31	403.91
**rs13737**	15	PTPN9	0.049	0.007	5.60E-10	118.60
**rs1377807**	17	ZZEF1	0.049	0.007	4.20E-13	139.08
**rs138337556**	5	SLCO6A1	0.445	0.077	4.70E-09	17.49
**rs140242150**	10	TCF7L2	0.307	0.056	2.20E-08	31.58
**rs1412234**	9	LINGO2	0.039	0.007	1.90E-10	89.63
**rs141521721**	11	PDE3B	0.122	0.020	2.70E-08	27.65
**rs1421085**	16	FTO	0.122	0.007	3.10E-84	982.82
**rs1426371**	12	WSCD2	0.049	0.007	8.20E-12	125.10
**rs145678014**	11	QSER1	0.104	0.016	2.00E-10	49.20
**rs145904381**	1	FAM63A	0.174	0.030	2.60E-08	18.94
**rs146886108**	5	ANKH	0.344	0.049	7.80E-13	45.32
**rs149364428**	8	CPQ	0.239	0.034	1.80E-12	31.53
**rs1493694**	1	NOTCH2	0.086	0.009	2.70E-16	152.51
**rs1531583**	4	PCGF3	0.122	0.016	3.50E-14	83.76
**rs1561927**	8	PVT1	0.039	0.007	1.50E-09	81.18
**rs1562396**	7	KLF14	0.058	0.007	9.90E-18	198.25
**rs1580278**	4	SLC9B1	0.039	0.005	2.20E-10	143.65
**rs1641523**	17	ATP1B2	0.049	0.007	1.20E-10	159.37
**rs17013314**	3	UBE2E2	0.104	0.018	8.40E-09	33.14
**rs1708302**	7	JAZF1	0.095	0.007	1.10E-48	611.10
**rs17122772**	14	SLC7A7	0.039	0.007	1.60E-08	72.94
**rs17168486**	7	DGKB	0.068	0.007	2.30E-17	184.84
**rs17250977**	5	ANKH	0.113	0.016	2.00E-11	58.09
**rs17261179**	5	ITGA1	0.039	0.007	1.30E-08	102.81
**rs17522122**	14	AKAP6	0.039	0.005	3.20E-09	143.65
**rs17684074**	18	WDR7	0.039	0.007	2.90E-08	79.25
**rs17689007**	8	MSRA	0.039	0.005	2.50E-09	143.65
**rs177045**	10	NEUROG3	0.068	0.007	6.60E-18	272.53
**rs17772814**	8	CASC11	0.077	0.014	5.40E-10	59.08
**rs17791513**	9	TLE4	0.095	0.012	3.10E-14	92.81
**rs17802463**	2	DTNB	0.039	0.005	2.90E-08	113.66
**rs17819328**	3	PPARG	0.058	0.007	4.80E-16	223.31
**rs1783541**	11	MAP3K11	0.058	0.007	2.00E-14	145.76

**rs17836088**	14	NRXN3	0.058	0.010	6.70E-14	109.43
**rs1800574**	12	HNF1A	0.131	0.020	1.70E-12	47.33
**rs1800961**	20	HNF4A	0.166	0.017	2.30E-22	117.99
**rs1801645**	22	PIM3	0.039	0.007	1.50E-08	83.03
**rs184509201**	10	TCF7L2	0.191	0.025	1.20E-13	54.21
**rs184660829**	1	DENND2C	2.086	0.375	2.50E-08	4.40
**rs2028150**	2	CEP68	0.049	0.007	2.30E-12	156.06
**rs2066827**	12	CDKN1B	0.049	0.007	4.20E-08	118.60
**rs2102278**	4	USP46	0.039	0.007	3.70E-08	89.63
**rs2197973**	12	USP44	0.039	0.007	3.60E-08	102.31
**rs2237895**	11	KCNQ1	0.113	0.007	6.00E-52	848.22
**rs2237897**	11	KCNQ1	0.207	0.017	8.40E-32	226.98
**rs2238689**	19	GIPR	0.039	0.005	5.40E-09	140.48
**rs2249105**	2	CEP68	0.095	0.012	2.20E-14	332.41
**rs2258238**	12	HMGA2	0.095	0.012	4.50E-21	128.31
**rs2268078**	20	RALY	0.039	0.007	2.30E-10	92.43
**rs2272163**	3	ROBO2	0.039	0.007	9.60E-09	97.04
**rs2280141**	10	PLEKHA1	0.049	0.007	1.40E-13	162.30
**rs2283220**	11	KCNQ1	0.049	0.007	1.40E-09	139.08
**rs2307111**	5	POC5	0.049	0.007	2.10E-16	154.69
**rs231349**	11	KCNQ1	0.068	0.012	2.30E-11	65.74
**rs231361**	11	KCNQ1	0.077	0.007	5.00E-25	308.98
**rs234853**	11	KCNQ1	0.077	0.009	6.80E-16	234.18
**rs243024**	2	BCL11A	0.058	0.005	2.50E-20	316.88
**rs2431115**	5	ANKRD55	0.039	0.007	3.90E-10	98.85
**rs2456530**	15	ONECUT1	0.058	0.010	5.40E-09	72.12
**rs2581787**	3	RFT1	0.039	0.007	2.40E-08	101.49
**rs2642588**	10	NEUROG3	0.049	0.007	2.20E-14	136.55
**rs2767036**	11	PDHX	0.039	0.007	3.30E-08	84.81
**rs2796441**	9	TLE1	0.068	0.007	4.40E-24	302.97
**rs279744**	5	ARL15	0.039	0.005	3.10E-08	123.35
**rs2800733**	6	SOGA3	0.049	0.007	6.00E-11	131.08
**rs2820446**	1	LYPLAL1	0.058	0.007	3.30E-16	187.59
**rs28505901**	9	GPSM1	0.086	0.009	6.70E-26	292.14
**rs2872246**	3	ABCC5	0.039	0.007	1.50E-08	101.94
**rs28819812**	4	PDGFC	0.039	0.007	2.20E-08	89.63
**rs291367**	1	GNG4	0.039	0.007	4.70E-10	96.01
**rs2925979**	16	CMIP	0.049	0.007	1.40E-14	136.55
**rs2972144**	2	IRS1	0.095	0.007	2.10E-46	563.38
**rs2982521**	6	MIR3668	0.049	0.007	1.30E-09	153.19
**rs3111316**	19	FARSA	0.049	0.007	6.30E-13	157.29

**rs3217792**	12	CCND2	0.113	0.011	2.60E-21	180.23
**rs3217860**	12	CCND2	0.049	0.007	3.90E-09	125.10
**rs329122**	5	PHF15	0.039	0.005	3.60E-09	141.34
**rs340874**	1	PROX1	0.068	0.007	1.60E-22	308.61
**rs34298980**	6	LRFN2	0.039	0.005	9.30E-10	144.17
**rs34454109**	20	TSHZ2	0.039	0.007	7.10E-09	72.94
**rs34584161**	13	RNF6	0.049	0.007	2.20E-10	118.60
**rs34715063**	15	RASGRP1	0.095	0.012	2.30E-19	150.56
**rs348330**	1	ABCB10	0.049	0.007	2.70E-14	149.81
**rs34855406**	17	MLX	0.049	0.007	2.30E-12	131.08
**rs34855922**	10	TCF7L2	0.049	0.007	5.50E-12	131.08
**rs34965774**	12	KSR2	0.058	0.010	2.00E-09	76.77
**rs35352848**	3	UBE2E2	0.068	0.010	1.30E-17	145.42
**rs35895680**	17	TTLL6	0.058	0.007	2.50E-15	198.25
**rs35913461**	2	TMEM18	0.058	0.010	1.60E-11	89.98
**rs35999103**	2	PABPC1P2	0.049	0.010	9.70E-09	61.17
**rs362307**	4	HTT	0.077	0.012	1.10E-09	68.93
**rs3751837**	16	CLUAP1	0.039	0.007	1.40E-08	70.68
**rs3768321**	1	MACF1	0.086	0.007	2.60E-26	320.53
**rs3772071**	2	RBMS1	0.049	0.007	1.20E-11	133.88
**rs3774723**	3	PSMD6	0.068	0.010	1.60E-13	117.81
**rs3798519**	6	TFAP2B	0.058	0.010	2.60E-12	94.12
**rs3802177**	8	SLC30A8	0.104	0.007	1.10E-55	626.88
**rs3810291**	19	ZC3H4	0.049	0.007	8.90E-12	143.76
**rs3811978**	5	ITGA1	0.058	0.007	7.70E-11	128.54
**rs3845281**	5	ANKH	0.077	0.009	2.30E-11	112.39
**rs3887925**	3	ST6GAL1	0.068	0.007	3.10E-22	309.99
**rs39328**	7	RELN	0.039	0.007	3.70E-08	100.95
**rs3934712**	5	EBF1	0.049	0.010	3.20E-08	75.50
**rs4148856**	12	MPHOSPH9	0.049	0.010	1.70E-10	78.10
**rs4238013**	12	CCND2	0.058	0.007	3.20E-11	151.14
**rs4279506**	7	IGF2BP3	0.058	0.010	4.80E-08	151.71
**rs4281707**	16	FTO	0.039	0.005	3.20E-10	143.24
**rs429358**	19	TOMM40/APOE	0.077	0.009	2.60E-18	159.23
**rs4457053**	5	ZBED3	0.058	0.007	8.40E-18	191.32
**rs465002**	5	ANKRD55	0.104	0.007	6.10E-38	563.83
**rs4686471**	3	LPP	0.058	0.007	1.70E-20	216.75
**rs4688760**	3	RBM6	0.039	0.007	3.50E-10	89.63
**rs4709746**	6	QKI	0.058	0.010	5.80E-09	72.12
**rs474513**	6	SLC22A3	0.039	0.005	8.10E-10	143.94
**rs4776970**	15	MAP2K5	0.039	0.005	5.00E-09	132.86
**rs4804833**	19	MAP2K7	0.049	0.007	7.70E-13	154.69

**rs4810426**	20	HNF4A	0.086	0.012	3.10E-17	114.38
**rs4923543**	11	METTL15	0.039	0.007	4.50E-08	91.07
**rs4925109**	17	RAI1	0.049	0.007	2.80E-12	141.49
**rs4929965**	11	INS/IGF2	0.068	0.007	4.00E-26	295.08
**rs4932265**	15	AP3S2	0.068	0.007	4.20E-20	246.85
**rs4946812**	6	BEND3	0.039	0.005	8.20E-09	127.50
**rs4976033**	5	PIK3R1	0.049	0.007	1.00E-09	157.29
**rs4977213**	8	BOP1	0.049	0.007	9.10E-14	153.19
**rs505922**	9	ABO	0.049	0.007	3.90E-12	143.76
**rs5213**	11	KCNJ11	0.068	0.007	3.50E-27	288.56
**rs523288**	18	MC4R	0.049	0.007	7.60E-13	118.60
**rs528350911**	15	WDR72	0.239	0.042	2.10E-08	25.52
**rs536643418**	10	TCF7L2	0.405	0.073	2.60E-08	42.16
**rs539515**	1	SEC16B	0.049	0.007	1.60E-10	104.03
**rs555402748**	6	SLC25A51P1	1.3	0.239	4.60E-08	5.36
**rs555759341**	11	INS/IGF2	0.322	0.059	3.60E-08	16.24
**rs55653563**	9	ZNF169	0.039	0.007	2.20E-09	81.18
**rs562386202**	2	DDX18	1.163	0.213	4.20E-08	7.22
**rs56337234**	4	MAEA	0.058	0.007	8.60E-18	227.78
**rs56348580**	12	HNF1A	0.049	0.007	2.30E-13	139.08
**rs569511541**	17	KIF2B	2.032	0.358	1.50E-08	4.37
**rs57235767**	11	MTNR1B	0.039	0.007	5.90E-10	84.81
**rs57327348**	8	XKR6	0.039	0.010	4.50E-08	49.47
**rs5758223**	22	EP300	0.039	0.005	3.80E-08	116.25
**rs576674**	13	KL	0.049	0.007	8.30E-10	91.74
**rs58432198**	1	FAF1	0.068	0.010	2.10E-10	92.56
**rs58730668**	4	ACSL1	0.068	0.010	1.30E-13	105.53
**rs59944054**	20	TCEA2	0.058	0.010	1.50E-08	116.31
**rs601945**	6	MHC	0.058	0.010	4.70E-08	94.12
**rs60276348**	17	ACE	0.049	0.010	2.60E-08	54.80
**rs6063048**	20	EYA2	0.049	0.007	2.20E-11	128.16
**rs62007683**	14	MARK3	0.039	0.007	3.10E-08	93.70
**rs62080313**	18	COMMD9	0.058	0.010	1.00E-08	67.34
**rs62107261**	2	TMEM18	0.113	0.016	3.80E-12	71.86
**rs62271373**	3	TSC22D2	0.086	0.014	1.00E-09	56.48
**rs62492368**	7	AOC1	0.049	0.007	1.10E-10	139.08
**rs6458354**	6	VEGFA	0.049	0.007	2.10E-12	133.88

**rs6459733**	7	MNX1	0.058	0.005	2.40E-17	282.04
**rs649961**	3	SLC12A8	0.039	0.005	9.90E-10	143.65
**rs6518681**	22	MTMR3/ASCC2	0.086	0.012	1.10E-12	95.69
**rs6545714**	2	BNIPL	0.039	0.007	8.90E-09	97.99
**rs6600191**	16	ITFG3	0.058	0.007	9.30E-13	128.54
**rs6708643**	2	THADA	0.039	0.007	3.90E-08	102.97
**rs67232546**	11	ETS1	0.058	0.007	1.30E-11	151.14
**rs67254669**	11	KCNJ11	0.637	0.111	1.10E-08	7.61
**rs6780171**	3	IGF2BP2	0.131	0.009	9.00E-56	773.72
**rs6821438**	4	SMARCAD1	0.039	0.007	4.00E-11	102.60
**rs6884702**	5	MRPS30	0.039	0.007	1.50E-10	97.99
**rs6885132**	5	ANKH	0.068	0.012	1.70E-08	65.74
**rs6976111**	7	CTTNBP2	0.039	0.007	1.20E-08	88.10
**rs7022807**	9	HAUS6	0.039	0.005	2.70E-10	138.40
**rs702634**	5	ARL15	0.049	0.007	7.70E-14	139.08
**rs7115753**	11	CRY2	0.039	0.005	3.80E-09	142.73
**rs7124681**	11	CELF1	0.039	0.005	5.10E-09	139.50
**rs71372253**	17	NF1	0.077	0.012	4.40E-08	52.82
**rs7178762**	15	USP3	0.039	0.005	5.40E-10	143.24
**rs718314**	12	ITPR2	0.049	0.007	8.40E-11	121.91
**rs7222481**	17	GLP2R	0.039	0.005	1.40E-08	125.48
**rs7240767**	18	LAMA1	0.039	0.007	1.60E-08	97.04
**rs7249758**	19	UHRF1	0.049	0.010	3.40E-09	72.82
**rs72631105**	10	WDR11	0.058	0.010	3.70E-09	98.14
**rs72802342**	16	BCAR1	0.157	0.013	4.00E-32	264.58
**rs72926932**	18	TCF4	0.086	0.012	1.00E-14	85.99
**rs73226260**	12	HNF1A	0.122	0.018	5.90E-11	45.61
**rs738408**	22	PNPLA3	0.049	0.010	1.40E-10	80.60
**rs74452128**	18	MC4R	0.14	0.022	1.00E-09	33.10
**rs74653713**	3	MBNL1	0.095	0.016	1.20E-08	41.06
**rs75253922**	19	INSR	0.049	0.007	2.70E-08	100.06
**rs76263492**	3	CACNA2D3	0.086	0.016	6.30E-09	41.62
**rs7629630**	3	EGFEM1P	0.049	0.010	2.50E-08	54.80
**rs7645517**	3	ST6GAL1	0.077	0.014	2.50E-08	45.27
**rs76549217**	5	ANKH	0.131	0.020	3.00E-10	47.33
**rs7669833**	4	TMEM154	0.058	0.007	1.20E-14	187.59
**rs76895963**	12	CCND2	0.482	0.027	1.40E-69	319.77
**rs77136196**	6	HMGA1	0.104	0.021	1.60E-08	37.49
**rs7719891**	5	RASA1	0.039	0.007	2.40E-08	79.25
**rs77464186**	11	CENTD2/ARAP1	0.104	0.009	4.70E-33	306.25
**rs7756992**	6	CDKAL1	0.14	0.009	2.40E-88	814.31
**rs77864822**	12	RMST	0.077	0.014	1.10E-08	52.26

**rs78020297**	16	FTO	0.086	0.014	6.50E-09	47.56
**rs78408340**	5	PAM	0.385	0.038	2.10E-24	73.20
**rs7867635**	9	FOCAD	0.039	0.007	4.00E-08	99.64
**rs7903146**	10	TCF7L2	0.315	0.007	5.8E-447	5677.77
**rs79046683**	2	TMEM127	0.85	0.154	3.00E-08	2.67
**rs7918400**	10	TCF7L2	0.058	0.007	2.00E-15	227.41
**rs79687284**	1	PROX1	0.148	0.020	2.60E-16	60.41
**rs7978610**	12	ZNF664	0.239	0.042	2.00E-08	570.31
**rs7987740**	13	IRS2	0.039	0.007	4.00E-08	97.99
**rs8010382**	14	SMEK1	0.039	0.005	6.50E-09	140.48
**rs80147536**	2	THADA	0.122	0.011	2.70E-29	230.88
**rs8017808**	14	CLEC14A	0.039	0.007	2.10E-08	79.25
**rs8032939**	15	RASGRP1	0.058	0.007	3.50E-14	170.82
**rs8046545**	16	ATP2A1	0.039	0.007	1.90E-08	94.90
**rs8107974**	19	TM6SF2	0.095	0.012	3.30E-15	104.93
**rs862320**	16	NFAT5	0.039	0.007	3.90E-11	100.34
**rs878521**	7	GCK	0.058	0.007	1.90E-13	170.82
**rs917195**	7	CRHR2	0.049	0.007	4.20E-11	115.15
**rs9379084**	6	RREB1	0.104	0.012	3.30E-21	167.28
**rs9494624**	6	SLC35D3	0.039	0.007	6.10E-09	84.81
**rs9505097**	6	RREB1	0.049	0.010	8.60E-10	72.82
**rs9537803**	13	PCDH17	0.039	0.007	4.60E-08	83.03
**rs9563615**	13	SRGAP2D	0.049	0.007	6.40E-11	133.88
**rs963740**	13	DLEU1	0.039	0.005	2.10E-08	118.73
**rs9687832**	5	ANKRD55	0.077	0.009	1.70E-20	199.83
**rs9828772**	3	TMCC1	0.058	0.010	4.20E-08	57.39
**rs9860730**	3	ADAMTS9	0.058	0.007	4.90E-15	191.32
**rs9873618**	3	SLC2A2	0.068	0.007	4.80E-21	257.87
**rs9957145**	18	GRP	0.049	0.010	8.10E-09	64.22
**Fasting glucose (35)**						47.79
**rs10747083**	12	P2RX2	0.013	0.002	7.57E-09	13.83
**rs10811661**	9	CDKN2B	0.024	0.003	5.65E-18	20.67
**rs10830963**	11	MTNR1B	0.078	0.002	0.00E+00	458.43
**rs10885122**	10	ADRA2A	0.027	0.003	6.32E-17	18.72
**rs11071657**	15	VPS13C/C2CD4A/B	0.01	0.002	2.96E-07	8.50
**rs11558471**	8	SLC30A8	0.029	0.002	7.80E-37	66.77
**rs11603334**	11	ARAP1	0.019	0.003	1.12E-11	12.39
**rs11605924**	11	CRY2	0.02	0.002	3.93E-19	36.47
**rs11619319**	13	PDX1	0.019	0.002	1.33E-15	23.32
**rs11708067**	3	ADCY5	0.023	0.003	1.30E-18	21.34

**rs11715915**	3	AMT	0.012	0.002	4.90E-08	11.43
**rs11920090**	3	SLC2A2	0.026	0.003	8.56E-18	19.79
**rs16913693**	9	IKBKAP	0.043	0.007	3.51E-11	5.61
**rs174550**	11	FADS1	0.019	0.002	1.34E-17	29.55
**rs17762454**	6	RREB1	0.014	0.002	9.57E-09	13.75
**rs2191349**	7	DGKB/TMEM195	0.029	0.002	0.00E+00	76.45
**rs2657879**	12	GLS2	0.016	0.003	3.94E-08	9.19
**rs340874**	1	PROX1	0.013	0.002	4.08E-10	15.39
**rs3783347**	14	WARS	0.017	0.003	1.32E-10	11.66
**rs3829109**	9	LOC728489	0.017	0.003	1.13E-10	14.47
**rs4506565**	10	TCF7L2	0.021	0.002	3.95E-19	33.78
**rs4607517**	7	GCK	0.057	0.003	0.00E+00	106.25
**rs4869272**	5	PCSK1	0.018	0.002	1.02E-15	25.28
**rs560887**	2	G6PC2	0.071	0.002	0.00E+00	387.20
**rs576674**	13	KL	0.017	0.003	2.26E-08	8.96
**rs6072275**	20	TOP1	0.016	0.003	1.66E-08	8.37
**rs6113722**	20	FOXA2	0.035	0.005	2.49E-11	6.86
**rs6943153**	7	GRB10	0.015	0.002	1.63E-12	18.42
**rs7651090**	3	IGF2BP2	0.013	0.002	1.75E-08	13.18
**rs7708285**	5	ZBED3	0.015	0.003	1.20E-08	10.78
**rs780094**	2	GCKR	0.027	0.002	2.58E-37	63.28
**rs7867224**	9	GLIS3	0.013	0.002	3.90E-09	15.40
**rs7944584**	11	MADD	0.023	0.002	4.82E-22	38.04
**rs9368222**	6	CDKAL1	0.014	0.002	1.00E-09	14.41
**rs983309**	8	PPP1R3B	0.026	0.003	6.29E-15	17.36
**Fasting insulin (18)**						16.52
**rs10195252**	2	GRB14	0.016	0.003	4.87E-10	15.06
**rs1167800**	7	HIP1	0.016	0.003	2.61E-09	15.46
**rs1421085**	16	FTO	0.02	0.003	1.87E-15	23.70
**rs1530559**	2	YSK4	0.015	0.003	3.37E-08	13.66
**rs17036328**	3	PPARG	0.021	0.003	3.59E-12	12.91
**rs2126259**	8	PPP1R3B	0.024	0.003	3.30E-13	13.71
**rs2745353**	6	RSPO3	0.014	0.002	5.48E-09	17.87
**rs2943645**	2	IRS1	0.019	0.002	2.26E-19	30.70
**rs3822072**	4	FAM13A1	0.012	0.002	1.80E-08	13.11
**rs459193**	5	ANKRD55	0.015	0.002	1.15E-10	16.18
**rs4846565**	1	LYPLAL1	0.013	0.002	1.76E-09	13.63
**rs4865796**	5	ARL15	0.015	0.003	2.09E-08	12.10

**rs6912327**	6	C6orf107	0.017	0.003	2.26E-08	11.24
**rs731839**	19	PEPD	0.015	0.003	5.13E-12	12.28
**rs780094**	2	GCKR	0.019	0.002	7.06E-14	31.33
**rs7903146**	10	TCF7L2	0.018	0.003	6.13E-11	15.88
**rs860598**	12	IGF1	0.018	0.003	1.64E-08	11.12
**rs974801**	4	TET2	0.014	0.002	3.27E-11	16.84
**HbA_{1c} (38)**						17.71
**rs1046896**	17	FN3KRP	0.028	0.002	4.00E-64	65.91
**rs10774625**	12	ATXN2	0.0088	0.003	1.00E-08	3.98
**rs10823343**	10	HK1	0.0325	0.003	2.00E-55	44.91
**rs10830963**	11	MTNR1B	0.0196	0.002	2.00E-23	23.23
**rs11248914**	16	FAM234A	0.0142	0.002	3.00E-14	13.42
**rs11558471**	8	SLC30A8/LOC105375716	0.015	0.002	1.00E-19	19.19
**rs11603334**	11	ARAP1	0.012	0.021	7.00E-09	0.62
**rs11708067**	3	ADCY5	0.0132	0.019	1.00E-12	1.02
**rs11964178**	6	CCDC162P	0.0096	0.016	6.00E-10	0.85
**rs12621844**	2	RN7SKP224	0.0099	0.018	2.00E-08	0.78
**rs13134327**	4	FREM3	0.0131	0.017	3.00E-15	1.31
**rs1558902**	16	FTO	0.0103	0.019	3.00E-08	0.81
**rs17509001**	2	ATAD2B	0.0181	0.002	2.00E-15	11.29
**rs17533903**	19	MYO9B	0.015	0.002	5.00E-12	12.06
**rs17747324**	10	TCF7L2	0.0149	0.002	6.00E-11	10.78
**rs1800562**	6	HFE/HFE-AS1	0.0398	0.003	5.00E-28	22.43
**rs198846**	6	H1-6	0.0218	0.002	1.00E-23	17.47
**rs2110073**	12	PHB2	0.0153	0.003	4.00E-08	4.84
**rs2383208**	9	CDKN2B-AS1	0.0142	0.002	7.00E-12	9.43
**rs2408955**	12	PFKM/SENP1	0.0124	0.002	1.00E-15	14.46
**rs267738**	1	CERS2	0.0111	0.019	3.00E-09	0.69
**rs3782123**	11	BET1L	0.0126	0.020	2.00E-10	1.03
**rs3824065**	7	YKT6	0.0192	0.002	4.00E-35	36.09
**rs4607517**	7	GCK	0.0306	0.002	9.00E-38	39.16
**rs4745982**	10	HK1	0.0954	0.006	3.00E-65	107.34
**rs4820268**	22	TMPRSS6	0.0162	0.002	1.00E-22	21.66
**rs560887**	2	G6PC2	0.0284	0.002	1.00E-58	57.91
**rs579459**	9	Y_RNA/ABO	0.0107	0.003	9.00E-09	3.55
**rs592423**	6	ATP5PBP6/LINC01625	0.0091	0.002	4.00E-08	7.27
**rs6474359**	8	ANK1	0.0436	0.005	2.00E-16	9.44
**rs7040409**	9	PCNPP2	0.0284	0.004	3.00E-14	11.74
**rs7616006**	3	SYN2/GSTM5P1	0.0103	0.003	5.00E-10	4.69
**rs7756992**	6	CDKAL1	0.0123	0.002	3.00E-12	10.27
**rs8192675**	3	SLC2A2	0.0112	0.002	1.00E-11	9.49

**rs837763**	16	LOC107984842	0.0172	0.002	2.00E-28	28.40
**rs857691**	1	SPTA1	0.0193	0.002	4.00E-25	24.47
**rs9604573**	13	GAS6/GAS6-AS1	0.0101	0.002	1.00E-08	6.74
**rs9914988**	17	ERAL1/FAM222B	0.0131	0.002	3.00E-11	9.86

Table S3 Definition and sources for CVDs in FinnGen.

CVDs	No. of cases	ICD-10 diagnosis for the first occurance
**Myocardial infarction**	4065	I21, I22
**Heart failure**	8016	I11.0, I13.0, I13.2, I50
**Ischemic heart disease**	10739	I20-I25
**Coronary atherosclerosis**	7661	I24, I25, T82.2, Z95.1
**Major coronary heart disease event**	7123	I20.0, I21, I22; Including revascularizations
**Peripheral artery disease**	2398	E10.5, E10.5+I79.2, E11.5, E11.5, I79.2, E12.5, E13.5, E14.5, I70.2, I73.9
**Essential hypertension**	22142	I10
**Stroke**	7144	I60-I64, G45
**Ischaemic stroke**	4026	I63, I64
**Intracerebral haemorrhage**	1149	I61
**Atrial fibrillation and fluttering**	7244	I48
**Cardiovascular mortality**	3480	I00-I02, I05-I09, I10-I15, I20-I25, I26-I28, I30-I52, R96, R98, R99

Table S4 The causal effect of genetically predicted type 2 diabetes mellitus on outcomes in different studies.

ID of outcome	Outcome	Methods	No. of SNPs	Beta	SE	*p* value	OR	LCI	UCI	Heterogeneity *p* value	*p* value for intercept	*p* value for MR-PRESSO	Distortion test *p* value
**ukb-d-I9_K_CARDIAC**	Cardiovascular mortality	MR Egger	273	0.000	0.000	0.130	1.000	1.000	1.001	0.367	0.399	0.379	

		Weighted median	273	0.001	0.000	**0.017**	1.001	1.000	1.001				
		Inverse variance weighted	273	0.001	0.000	**0.000**	1.001	1.000	1.001	0.372			
		Simple mode	273	0.001	0.001	**0.038**	1.001	1.000	1.003				
		Weighted mode	273	0.001	0.000	0.055	1.001	1.000	1.001				
		MR-PRESSO	273	0.001	0.000	**0.000**	1.001	1.000	1.001				
**ieu-a-798**	Myocardial infarction	MR Egger	268	0.021	0.030	0.485	1.021	0.963	1.084	0.000	0.000	<0.001	0.687
		Weighted median	268	0.079	0.024	**0.001**	1.083	1.032	1.135				
		Inverse variance weighted	268	0.118	0.016	**0.000**	1.126	1.092	1.161	0.000			
		Simple mode	268	0.095	0.055	0.087	1.099	0.987	1.225				
		Weighted mode	268	0.069	0.025	**0.007**	1.071	1.019	1.125				
		MR-PRESSO	268	0.120	0.016	**0.000**	1.127	1.094	1.162				
**ukb-d-I9_INTRACRA**	Intracranial haemorrhage	MR Egger	271	0.000	0.000	0.213	1.000	0.999	1.000	0.099	0.065	0.121	
		Weighted median	271	0.000	0.000	0.860	1.000	0.999	1.001				
		Inverse variance weighted	271	-0.001	0.001	0.172	0.999	0.998	1.000	0.081			
		Simple mode	271	0.000	0.000	0.493	1.000	1.000	1.000				
		Weighted mode	271	0.000	0.000	0.177	1.000	0.999	1.000				
		MR-PRESSO	271	0.000	0.000	0.489	1.000	1.000	1.000				
**ebi-a-GCST009541**	Heart failure	MR Egger	232	-0.014	0.025	0.578	0.986	0.939	1.036	0.000	0.001	<0.001	0.511
		Weighted median	232	0.014	0.019	0.458	1.014	0.977	1.053				
		Inverse variance weighted	232	0.059	0.013	**0.000**	1.061	1.035	1.088	0.000			
		Simple mode	232	0.065	0.044	0.141	1.067	0.979	1.163				
		Weighted mode	232	0.014	0.019	0.464	1.014	0.977	1.054				
		MR-PRESSO	232	0.066	0.011	**0.000**	1.068	1.045	1.092				

**ieu-a-7**	Ischemic heart disease	MR Egger	269	0.031	0.028	0.276	1.031	0.976	1.090	0.000	0.000	<0.001	0.586
		Weighted median	269	0.106	0.023	**0.000**	1.111	1.063	1.162				
		Inverse variance weighted	269	0.128	0.015	**0.000**	1.137	1.105	1.170	0.000			
		Simple mode	269	0.179	0.051	**0.001**	1.196	1.082	1.323				
		Weighted mode	269	0.097	0.026	**0.000**	1.102	1.046	1.161				
		MR-PRESSO	269	0.125	0.015	**0.000**	1.134	1.102	1.167				
**ukb-d-I9_CORATHER**	Coronary atherosclerosis	MR Egger	277	0.003	0.001	**0.008**	1.003	1.001	1.006	0.000	0.096	<0.001	0.762
		Weighted median	277	0.005	0.001	**0.000**	1.005	1.004	1.007				
		Inverse variance weighted	277	0.005	0.001	**0.000**	1.005	1.004	1.007	0.000			
		Simple mode	277	0.008	0.003	**0.002**	1.008	1.003	1.013				
		Weighted mode	277	0.004	0.001	**0.000**	1.004	1.002	1.006				
		MR-PRESSO	277	0.005	0.001	**0.000**	1.005	1.004	1.007				
**ukb-d-I9_CHD**	Major coronary heart disease event	MR Egger	276	0.002	0.001	0.101	1.002	1.000	1.004	0.000	0.047	<0.001	0.96
		Weighted median	276	0.003	0.001	**0.000**	1.003	1.002	1.005				
		Inverse variance weighted	276	0.003	0.001	**0.000**	1.003	1.002	1.004	0.000			
		Simple mode	276	0.003	0.002	0.125	1.003	0.999	1.006				
		Weighted mode	276	0.003	0.001	**0.000**	1.003	1.001	1.004				
		MR-PRESSO	276	0.003	0.001	**0.000**	1.003	1.002	1.004				
**bbj-a-144**	Peripheral artery disease	MR Egger	217	0.120	0.051	**0.020**	1.128	1.020	1.247	0.059	0.075	0.039	NA*
		Weighted median	217	0.115	0.053	**0.030**	1.122	1.011	1.245				
		Inverse variance weighted	217	0.195	0.030	**0.000**	1.215	1.145	1.289	0.047			

		Simple mode	217	0.099	0.110	0.366	1.105	0.891	1.370				
		Weighted mode	217	0.099	0.054	0.069	1.105	0.993	1.229				
		MR-PRESSO	217	0.190	0.029	**0.000**	1.209	1.141	1.280				
**ukb-b-12493**	Essential hypertension	MR Egger	276	0.002	0.002	0.431	1.002	0.997	1.006	0.000	0.000	<0.001	0.953
		Weighted median	276	0.006	0.002	**0.000**	1.006	1.003	1.009				
		Inverse variance weighted	276	0.012	0.001	**0.000**	1.012	1.010	1.014	0.000			
		Simple mode	276	0.006	0.004	0.122	1.006	0.998	1.014				
		Weighted mode	276	0.005	0.001	**0.000**	1.005	1.003	1.008				
		MR-PRESSO	276	0.012	0.001	**0.000**	1.012	1.010	1.014				
**ebi-a-GCST006906**	Stroke	MR Egger	268	0.050	0.023	**0.033**	1.051	1.004	1.100	0.000	0.224	0.001	0.619
		Weighted median	268	0.049	0.018	**0.006**	1.050	1.014	1.087				
		Inverse variance weighted	268	0.074	0.012	**0.000**	1.077	1.052	1.102	0.000			
		Simple mode	268	0.047	0.041	0.247	1.048	0.968	1.136				
		Weighted mode	268	0.051	0.020	**0.013**	1.052	1.011	1.095				
		MR-PRESSO	268	0.074	0.011	**0.000**	1.077	1.053	1.101				
**ebi-a-GCST006908**	Ischemic stroke	MR Egger	269	0.063	0.024	**0.010**	1.065	1.015	1.118	0.001	0.337	0.005	0.88
		Weighted median	269	0.067	0.020	**0.001**	1.069	1.028	1.112				
		Inverse variance weighted	269	0.084	0.012	**0.000**	1.087	1.061	1.114	0.001			
		Simple mode	269	0.063	0.051	0.212	1.065	0.965	1.176				
		Weighted mode	269	0.067	0.022	**0.002**	1.069	1.024	1.116				
		MR-PRESSO	269	0.083	0.012	**0.000**	1.087	1.061	1.112				
**ebi-a-GCST006414**	Atrial fibrillation and fluttering	MR Egger	283	-0.008	0.022	0.715	0.992	0.949	1.036	0.000	0.172	<0.001	0.097
		Weighted median	283	-0.022	0.015	0.147	0.978	0.950	1.008				

		Inverse variance weighted	283	0.018	0.012	0.117	1.018	0.995	1.042	0.000			
		Simple mode	283	-0.050	0.044	0.253	0.951	0.873	1.036				
		Weighted mode	283	-0.039	0.018	**0.027**	0.962	0.929	0.995				
		MR-PRESSO	283	0.018	0.011	0.121	1.018	0.995	1.041				
**ukb-d-30760_irnt**	HDL cholesterol	MR Egger	277	-0.016	0.008	0.051	0.985	0.969	1.000	0.000	0.010	<0.001	0.106
		Weighted median	277	-0.013	0.002	**0.000**	0.987	0.983	0.991				
		Inverse variance weighted	277	-0.033	0.004	**0.000**	0.967	0.960	0.975	0.000			
		Simple mode	277	-0.012	0.006	**0.049**	0.988	0.977	1.000				
		Weighted mode	277	-0.009	0.002	**0.000**	0.991	0.987	0.995				
		MR-PRESSO	277	-0.034	0.004	**0.000**	0.967	0.959	0.974				
**ukb-d-30780_irnt**	LDL cholesterol	MR Egger	277	-0.019	0.019	0.327	0.982	0.946	1.019	0.000	0.690	<0.001	0.013
		Weighted median	277	-0.015	0.004	**0.000**	0.985	0.977	0.993				
		Inverse variance weighted	277	-0.025	0.010	**0.010**	0.975	0.957	0.994	0.000			
		Simple mode	277	-0.017	0.012	0.172	0.983	0.960	1.007				
		Weighted mode	277	-0.017	0.006	**0.006**	0.983	0.972	0.995				
		MR-PRESSO	277	-0.034	0.004	**0.000**	0.967	0.959	0.974				
**ukb-d-30870_irnt**	Triglycerides	MR Egger	277	0.043	0.026	0.098	1.044	0.992	1.097	0.000	0.091	<0.001	0.118
		Weighted median	277	0.052	0.006	**0.000**	1.054	1.041	1.067				
		Inverse variance weighted	277	0.080	0.013	**0.000**	1.083	1.056	1.112	0.000			
		Simple mode	277	0.059	0.013	**0.000**	1.060	1.033	1.088				

		Weighted mode	277	0.042	0.007	**0.000**	1.043	1.029	1.057				
		MR-PRESSO	277	0.082	0.013	**0.000**	1.086	1.058	1.114				
**ukb-d-30630_irnt**	Apolipoprotein A	MR Egger	277	-0.008	0.005	0.137	0.992	0.982	1.002	0.000	0.021	<0.001	<0.001
		Weighted median	277	-0.006	0.001	**0.000**	0.994	0.992	0.997				
		Inverse variance weighted	277	-0.018	0.003	**0.000**	0.982	0.977	0.987	0.000			
		Simple mode	277	-0.006	0.003	0.107	0.994	0.988	1.001				
		Weighted mode	277	-0.006	0.002	**0.002**	0.994	0.991	0.998				
		MR-PRESSO	277	-0.018	0.003	**0.000**	0.982	0.977	0.987				
**ukb-d-30640_irnt**	Apolipoprotein B	MR Egger	277	-0.004	0.006	0.560	0.996	0.984	1.009	0.000	0.879	<0.001	0.081
		Weighted median	277	-0.001	0.002	0.524	0.999	0.996	1.002				
		Inverse variance weighted	277	-0.003	0.003	0.377	0.997	0.991	1.004	0.000			
		Simple mode	277	-0.006	0.004	0.188	0.994	0.986	1.003				
		Weighted mode	277	-0.006	0.002	**0.002**	0.994	0.991	0.998				
		MR-PRESSO	277	-0.003	0.003	0.416	0.997	0.991	1.004				
**ukb-d-30790_irnt**	Lipoprotein A	MR Egger	277	-0.030	0.029	0.299	0.971	0.917	1.027	0.000	0.215	<0.001	0.456
		Weighted median	277	-0.010	0.005	0.056	0.990	0.980	1.000				
		Inverse variance weighted	277	0.001	0.015	0.961	1.001	0.972	1.030	0.000			
		Simple mode	277	-0.003	0.011	0.776	0.997	0.975	1.019				
		Weighted mode	277	-0.003	0.006	0.606	0.997	0.985	1.009				
		MR-PRESSO	277	0.000	0.014	0.976	1.000	0.973	1.029				
**FinnGen Study**													

**finn-a-I9_K_CARDIAC**	Cardiovascular mortality	MR Egger	231	0.034	0.042	0.422	1.035	0.952	1.125	0.142	0.546	0.022	NA*
		Weighted median	231	0.062	0.037	0.091	1.064	0.990	1.143				
		Inverse variance weighted	231	0.056	0.022	**0.011**	1.058	1.013	1.104	0.148			
		Simple mode	231	0.014	0.077	0.861	1.014	0.871	1.179				
		Weighted mode	231	0.053	0.038	0.169	1.054	0.978	1.137				
		MR-PRESSO	231	0.058	0.021	**0.005**	1.060	1.018	1.104				
**finn-a-I9_MI**	Myocardial infarction	MR Egger	231	0.102	0.042	**0.016**	1.108	1.020	1.203	0.000	0.383	0.016	NA*
		Weighted median	231	0.096	0.031	**0.002**	1.101	1.036	1.171				
		Inverse variance weighted	231	0.134	0.022	**0.000**	1.143	1.095	1.193	0.000			
		Simple mode	231	0.154	0.076	**0.044**	1.167	1.005	1.355				
		Weighted mode	231	0.111	0.031	**0.000**	1.117	1.052	1.187				
		MR-PRESSO	231	-0.091	0.085	0.295	0.913	0.773	1.080				
**finn-a-I9_ICH**	Intracerebral hemorrhage	MR Egger	231	0.016	0.060	0.790	1.016	0.903	1.143	0.945	0.232	0.113	
		Weighted median	231	0.111	0.054	**0.041**	1.117	1.004	1.243				
		Inverse variance weighted	231	0.078	0.031	**0.013**	1.081	1.016	1.149	0.942			
		Simple mode	231	0.262	0.123	**0.035**	1.299	1.021	1.653				
		Weighted mode	231	0.100	0.064	0.122	1.105	0.974	1.254				
		MR-PRESSO	231	0.089	0.100	0.373	1.094	0.899	1.330				
**finn-a-HEARTFAIL**	Heart failure	MR Egger	231	-0.038	0.036	0.292	0.962	0.896	1.033	0.001	0.040	0.003	0.224
		Weighted median	231	-0.011	0.029	0.711	0.989	0.935	1.047				
		Inverse variance weighted	231	0.026	0.019	0.178	1.026	0.988	1.065	0.000			
		Simple mode	231	0.166	0.069	**0.018**	1.181	1.030	1.353				

		Weighted mode	231	-0.021	0.032	0.516	0.979	0.920	1.043			
		MR-PRESSO	231	0.048	0.037	0.203	1.049	0.975	1.129			
**finn-a-I9_ISCHHEART**	Ischemic heart disease	MR Egger	231	0.106	0.032	**0.001**	1.112	1.045	1.183	0.000	0.784	0.051
		Weighted median	231	0.094	0.023	**0.000**	1.099	1.050	1.150			
		Inverse variance weighted	231	0.114	0.016	**0.000**	1.120	1.085	1.157	0.000		
		Simple mode	231	0.067	0.052	0.197	1.069	0.966	1.183			
		Weighted mode	231	0.083	0.024	**0.001**	1.086	1.036	1.138			
		MR-PRESSO	231	0.114	0.031	**0.000**	1.121	1.054	1.192			
**finn-a-I9_CORATHER**	Coronary atherosclerosis	MR Egger	231	0.088	0.036	**0.016**	1.092	1.017	1.173	0.000	0.328	0.062
		Weighted median	231	0.078	0.027	**0.003**	1.082	1.026	1.140			
		Inverse variance weighted	231	0.118	0.019	**0.000**	1.126	1.085	1.168	0.000		
		Simple mode	231	0.101	0.062	0.104	1.106	0.980	1.248			
		Weighted mode	231	0.076	0.028	**0.007**	1.079	1.021	1.141			
		MR-PRESSO	231	0.128	0.036	**0.001**	1.136	1.058	1.220			
**finn-a-I9_CHD**	Major coronary heart disease event	MR Egger	231	0.082	0.037	**0.029**	1.085	1.009	1.168	0.000	0.509	0.130
		Weighted median	231	0.123	0.026	**0.000**	1.131	1.074	1.191			
		Inverse variance weighted	231	0.103	0.019	**0.000**	1.109	1.067	1.152	0.000		
		Simple mode	231	0.196	0.066	**0.003**	1.216	1.069	1.383			
		Weighted mode	231	0.108	0.031	**0.001**	1.114	1.048	1.184			
		MR-PRESSO	231	0.082	0.036	**0.023**	1.086	1.012	1.164			
**finn-a-I9_PAD**	Peripheral artery disease	MR Egger	231	0.113	0.049	**0.022**	1.120	1.017	1.232	0.000	0.093	0.073
		Weighted median	231	0.165	0.036	**0.000**	1.180	1.099	1.266			

		Inverse variance weighted	231	0.183	0.026	**0.000**	1.201	1.143	1.263	0.000			
		Simple mode	231	0.105	0.088	0.231	1.111	0.935	1.320				
		Weighted mode	231	0.134	0.037	**0.000**	1.143	1.063	1.230				
		MR-PRESSO	231	0.157	0.056	**0.006**	1.169	1.047	1.306				
**finn-a-I9_HYPTENSESS**	Essential hypertension	MR Egger	231	0.025	0.030	0.402	1.026	0.967	1.088	0.000	0.000	<0.001	0.842
		Weighted median	231	0.063	0.023	**0.006**	1.065	1.018	1.114				
		Inverse variance weighted	231	0.122	0.016	**0.000**	1.130	1.095	1.166	0.000			
		Simple mode	231	0.023	0.058	0.692	1.023	0.913	1.147				
		Weighted mode	231	0.017	0.020	0.397	1.017	0.978	1.059				
		MR-PRESSO	231	0.059	0.031	0.058	1.061	0.999	1.126				
**finn-a-C_STROKE**	Stroke	MR Egger	231	0.049	0.029	0.091	1.050	0.992	1.111	0.134	0.044	0.788	
		Weighted median	231	0.095	0.025	**0.000**	1.100	1.047	1.156				
		Inverse variance weighted	231	0.099	0.015	**0.000**	1.104	1.071	1.137	0.105			
		Simple mode	231	0.086	0.057	0.135	1.090	0.974	1.219				
		Weighted mode	231	0.112	0.034	**0.001**	1.119	1.047	1.196				
		MR-PRESSO	231	0.056	0.031	0.070	1.058	0.996	1.123				
**finn-a-I9_STR_EXH**	Ischemic stroke	MR Egger	231	0.033	0.034	0.346	1.033	0.966	1.105	0.139	0.306	0.404	
		Weighted median	231	0.078	0.030	**0.008**	1.081	1.020	1.146				
		Inverse variance weighted	231	0.063	0.018	**0.000**	1.065	1.028	1.103	0.138			
		Simple mode	231	0.122	0.066	0.065	1.129	0.993	1.284				
		Weighted mode	231	0.105	0.042	**0.013**	1.110	1.023	1.204				
		MR-PRESSO	231	0.038	0.041	0.354	1.039	0.959	1.125				
**finn-a-I9_AF**	Atrial fibrillation	MR Egger	231	-0.067	0.042	0.113	0.935	0.862	1.016	0.000	0.024	0.009	NA*

	and fluttering												
		Weighted median	231	-0.043	0.032	0.180	0.958	0.900	1.020				
		Inverse variance weighted	231	0.015	0.022	0.505	1.015	0.972	1.060	0.000			
		Simple mode	231	0.072	0.072	0.320	1.074	0.933	1.236				
		Weighted mode	231	-0.018	0.034	0.595	0.982	0.918	1.050				
		MR-PRESSO	231	0.035	0.043	0.418	1.036	0.952	1.127				
**The Global Lipids Genetics Consortium**													
**ieu-a-299**	HDL cholesterol	MR Egger	124	0.010	0.034	0.772	1.010	0.944	1.081	0.000	0.004	<0.001	0.019
		Weighted median	124	-0.008	0.011	0.460	0.992	0.971	1.013				
		Inverse variance weighted	124	-0.075	0.019	**0.000**	0.928	0.894	0.962	0.000			
		Simple mode	124	-0.029	0.021	0.171	0.971	0.932	1.012				
		Weighted mode	124	0.000	0.009	0.977	1.000	0.982	1.019				
		MR-PRESSO	124	-0.077	0.017	**0.000**	0.926	0.896	0.957				
**ieu-a-300**	LDL cholesterol	MR Egger	122	0.019	0.022	0.379	1.019	0.977	1.063	0.000	0.914	<0.001	0.554
		Weighted median	122	0.018	0.011	0.106	1.018	0.996	1.040				
		Inverse variance weighted	122	0.017	0.011	0.134	1.017	0.995	1.040	0.000			
		Simple mode	122	0.008	0.021	0.683	1.008	0.969	1.050				
		Weighted mode	122	0.018	0.010	0.054	1.019	1.000	1.038				
		MR-PRESSO	122	-0.077	0.017	**0.000**	0.926	0.896	0.957				
**ieu-a-302**	Triglycerides	MR Egger	123	-0.001	0.045	0.981	0.999	0.914	1.092	0.000	0.140	<0.001	0.442
		Weighted median	123	0.028	0.010	**0.008**	1.028	1.007	1.049				
		Inverse variance	123	0.056	0.024	**0.020**	1.058	1.009	1.109	0.000			

		weighted											
		Simple mode	123	0.031	0.023	0.180	1.032	0.986	1.080				
		Weighted mode	123	0.015	0.010	0.114	1.015	0.997	1.035				
		MR-PRESSO	123	0.022	0.010	**0.032**	1.023	1.002	1.044				

*No significant outliers

Table S5 The causal effect of genetically predicted FG on outcomes in different studies.

**ID of outcome**	**Outcome**	**Methods**	**No. of SNPs**	**Beta**	**SE**	***p* value**	**OR**	**LCI**	**UCI**	**Heterogeneity *p* value**	***p* value for intercept**	***p* value for MR-PRESSO**	**Distortion test *p* value**
**ukb-d-I9_K_CARDIAC**	Cardiovascular mortality	MR Egger	35	0.003	0.002	0.115	1.003	0.999	1.007	0.978	0.990	0.993	
		Weighted median	35	0.003	0.001	0.056	1.003	1.000	1.006				
		Inverse variance weighted	35	0.003	0.001	**0.004**	1.003	1.001	1.005	0.984			
		Simple mode	35	0.002	0.003	0.374	1.002	0.997	1.008				
		Weighted mode	35	0.003	0.001	0.057	1.003	1.000	1.006				
		MR-PRESSO	35	0.003	0.001	**0.000**	1.003	1.002	1.005				
**ieu-a-798**	Myocardial infarction	MR Egger	35	0.051	0.179	0.777	1.053	0.741	1.496	0.005	0.282	0.01	NA*
		Weighted median	35	0.208	0.115	0.071	1.231	0.982	1.544				
		Inverse variance weighted	35	0.214	0.100	**0.032**	1.239	1.018	1.508	0.004			
		Simple mode	35	0.127	0.197	0.525	1.135	0.771	1.672				
		Weighted mode	35	0.144	0.109	0.194	1.155	0.933	1.429				
		MR-PRESSO	35	0.214	0.100	**0.040**	1.239	1.018	1.508				
**ukb-d-I9_INTRACRA**	Intracranial haemorrhage	MR Egger	35	-0.001	0.002	0.480	0.999	0.995	1.002	0.700	0.891	0.718	
		Weighted median	35	-0.001	0.001	0.452	0.999	0.996	1.002				

		Inverse variance weighted	35	-0.001	0.001	0.140	0.999	0.997	1.000	0.635			
		Simple mode	35	-0.003	0.003	0.361	0.997	0.992	1.003				
		Weighted mode	35	-0.001	0.001	0.456	0.999	0.996	1.002				
		MR-PRESSO	35	-0.001	0.001	0.120	0.999	0.997	1.000				
**ebi-a-GCST009541**	Heart failure	MR Egger	35	0.093	0.170	0.590	1.097	0.786	1.531	0.009	0.597	0.011	NA*
		Weighted median	35	0.205	0.103	**0.047**	1.228	1.002	1.504				
		Inverse variance weighted	35	0.170	0.087	0.051	1.186	0.999	1.407	0.011			
		Simple mode	35	0.242	0.178	0.183	1.274	0.899	1.804				
		Weighted mode	35	0.213	0.096	**0.034**	1.237	1.025	1.493				
		MR-PRESSO	35	0.116	0.075	0.129	1.123	0.970	1.300				
**ieu-a-7**	Ischemic heart disease	MR Egger	35	0.115	0.174	0.514	1.122	0.798	1.576	0.000	0.389	0.707	
		Weighted median	35	0.234	0.097	**0.016**	1.264	1.045	1.529				
		Inverse variance weighted	35	0.241	0.096	**0.012**	1.272	1.054	1.536	0.000			
		Simple mode	35	0.154	0.185	0.411	1.166	0.812	1.675				
		Weighted mode	35	0.201	0.087	**0.028**	1.222	1.030	1.450				
		MR-PRESSO	35	0.241	0.096	**0.017**	1.272	1.054	1.536				
**ukb-d-I9_CORATHER**	Coronary atherosclerosis	MR Egger	35	0.013	0.008	0.128	1.013	0.997	1.030	0.000	0.932	<0.001	0.695
		Weighted median	35	0.018	0.005	**0.000**	1.019	1.008	1.029				
		Inverse variance weighted	35	0.014	0.005	**0.003**	1.014	1.005	1.023	0.000			
		Simple mode	35	0.021	0.011	0.067	1.021	0.999	1.043				
		Weighted mode	35	0.015	0.005	**0.002**	1.015	1.006	1.025				
		MR-PRESSO	35	0.016	0.004	**0.001**	1.016	1.007	1.025				

**ukb-d-I9_CHD**	Major coronary heart disease event	MR Egger	35	0.006	0.007	0.378	1.006	0.992	1.021	0.097	0.784	0.092	
		Weighted median	35	0.011	0.005	**0.020**	1.011	1.002	1.020				
		Inverse variance weighted	35	0.008	0.003	**0.017**	1.008	1.001	1.015	0.117			
		Simple mode	35	0.014	0.009	0.141	1.014	0.996	1.032				
		Weighted mode	35	0.010	0.006	0.079	1.010	0.999	1.022				
		MR-PRESSO	35	0.005	0.003	0.076	1.005	1.000	1.011				
**bbj-a-144**	Peripheral artery disease	MR Egger	32	-0.783	0.556	0.170	0.457	0.154	1.360	0.421	0.004	0.126	
		Weighted median	32	0.301	0.356	0.398	1.351	0.672	2.717				
		Inverse variance weighted	32	0.791	0.281	**0.005**	2.206	1.271	3.827	0.105			
		Simple mode	32	1.105	0.571	0.062	3.019	0.986	9.245				
		Weighted mode	32	0.469	0.402	0.253	1.598	0.726	3.517				
		MR-PRESSO	32	0.617	0.221	**0.009**	1.853	1.201	2.860				
**ukb-b-12493**	Essential hypertension	MR Egger	35	-0.008	0.018	0.680	0.992	0.957	1.029	0.000	0.426	<0.001	0.616
		Weighted median	35	0.004	0.008	0.626	1.004	0.989	1.019				
		Inverse variance weighted	35	0.005	0.009	0.547	1.005	0.988	1.023	0.000			
		Simple mode	35	-0.003	0.012	0.832	0.997	0.974	1.021				
		Weighted mode	35	0.003	0.007	0.651	1.003	0.989	1.017				
		MR-PRESSO	35	0.008	0.006	0.204	1.008	0.996	1.019				
**ebi-a-GCST006906**	Stroke	MR Egger	34	-0.158	0.175	0.373	0.854	0.607	1.202	0.001	0.030	<0.001	0.801
		Weighted median	34	-0.022	0.111	0.843	0.978	0.787	1.216				
		Inverse variance	34	0.172	0.103	0.095	1.187	0.970	1.453	0.000			

		weighted											
		Simple mode	34	-0.017	0.222	0.939	0.983	0.636	1.519				
		Weighted mode	34	-0.029	0.092	0.753	0.971	0.811	1.164				
		MR-PRESSO	34	0.172	0.103	0.105	1.187	0.970	1.453				
**ebi-a-GCST006908**	Ischemic stroke	MR Egger	35	-0.212	0.180	0.246	0.809	0.569	1.150	0.005	0.009	<0.001	0.399
		Weighted median	35	-0.064	0.127	0.616	0.938	0.732	1.203				
		Inverse variance weighted	35	0.206	0.109	0.058	1.228	0.993	1.520	0.000			
		Simple mode	35	0.013	0.255	0.959	1.013	0.615	1.671				
		Weighted mode	35	-0.045	0.102	0.659	0.956	0.783	1.166				
		MR-PRESSO	35	0.206	0.109	0.067	1.228	0.993	1.520				
**ebi-a-GCST006414**	Atrial fibrillation and fluttering	MR Egger	35	0.026	0.115	0.824	1.026	0.818	1.286	0.002	0.423	0.002	0.36
		Weighted median	35	-0.070	0.078	0.367	0.932	0.801	1.086				
		Inverse variance weighted	35	-0.052	0.064	0.420	0.950	0.837	1.077	0.003			
		Simple mode	35	-0.085	0.156	0.587	0.918	0.064	1.246				
		Weighted mode	35	0.004	0.075	0.956	1.004	0.866	1.164				
		MR-PRESSO	35	-0.052	0.064	0.426	0.950	0.837	1.077				
**ukb-d-30760_irnt**	HDL cholesterol	MR Egger	35	-0.026	0.080	0.749	0.974	0.833	1.140	0.000	0.521	<0.001	0.096
		Weighted median	35	0.013	0.012	0.286	1.013	0.989	1.038				
		Inverse variance weighted	35	0.020	0.038	0.608	1.020	0.946	1.099	0.000			
		Simple mode	35	0.003	0.024	0.898	1.003	0.957	1.052				
		Weighted mode	35	0.008	0.012	0.529	1.008	0.984	1.032				
		MR-PRESSO	35	0.002	0.010	0.861	1.002	0.983	1.021				

**ukb-d-30780_irnt**	LDL cholesterol	MR Egger	35	-0.050	0.108	0.650	0.952	0.769	1.177	0.000	0.899	<0.001	<0.001
		Weighted median	35	-0.010	0.021	0.640	0.990	0.951	1.032				
		Inverse variance weighted	35	-0.038	0.060	0.524	0.963	0.856	1.083	0.000			
		Simple mode	35	0.046	0.043	0.283	1.048	0.964	1.139				
		Weighted mode	35	0.001	0.019	0.969	1.001	0.963	1.039				
		MR-PRESSO	35	-0.038	0.060	0.528	0.963	0.856	1.083				
**ukb-d-30870_irnt**	Triglycerides	MR Egger	35	0.050	0.251	0.844	1.051	0.642	1.720	0.000	0.485	<0.001	<0.001
		Weighted median	35	0.125	0.028	**0.000**	1.133	1.073	1.196				
		Inverse variance weighted	35	-0.097	0.140	0.488	0.907	0.690	1.194	0.000			
		Simple mode	35	0.079	0.067	0.247	1.083	0.949	1.236				
		Weighted mode	35	0.101	0.021	**0.000**	1.106	1.061	1.153				
		MR-PRESSO	35	-0.097	0.140	0.493	0.907	0.690	1.194				
**ukb-d-30630_irnt**	Apolipoprotein A	MR Egger	35	-0.024	0.039	0.540	0.976	0.904	1.054	0.000	0.792	<0.001	0.452
		Weighted median	35	-0.017	0.007	**0.012**	0.983	0.970	0.996				
		Inverse variance weighted	35	-0.016	0.004	0.470	0.984	0.976	0.993	0.000			
		Simple mode	35	-0.017	0.014	0.235	0.983	0.957	1.011				
		Weighted mode	35	-0.011	0.006	0.082	0.989	0.977	1.001				
		MR-PRESSO	35	-0.016	0.022	0.474	0.984	0.944	1.027				
**ukb-d-30640_irnt**	Apolipoprotein B	MR Egger	35	-0.015	0.037	0.686	0.985	0.915	1.060	0.000	0.749	<0.001	0.051
		Weighted median	35	0.003	0.006	0.580	1.003	0.992	1.015				
		Inverse variance	35	-0.005	0.021	0.799	0.995	0.955	1.036	0.000			

		weighted										
		Simple mode	35	0.011	0.012	0.369	1.011	0.987	1.036			
		Weighted mode	35	0.004	0.005	0.385	1.004	0.995	1.014			
		MR-PRESSO	35	-0.005	0.021	0.801	0.995	0.955	1.036			
**ukb-d-30790_irnt**	Lipoprotein A	MR Egger	35	-0.001	0.041	0.972	0.999	0.921	1.083	0.040	0.624	0.06
		Weighted median	35	-0.033	0.030	0.277	0.968	0.913	1.027			
		Inverse variance weighted	35	-0.018	0.023	0.423	0.982	0.939	1.027	0.048		
		Simple mode	35	-0.077	0.054	0.159	0.926	0.833	1.028			
		Weighted mode	35	-0.019	0.027	0.497	0.982	0.931	1.035			
		MR-PRESSO	35	-0.018	0.023	0.428	0.982	0.939	1.027			
**FinnGen Study**												
**finn-a-I9_K_CARDIAC**	Cardiovascular mortality	MR Egger	17	0.287	0.497	0.572	1.333	0.503	3.530	0.546	0.927	0.641
		Weighted median	17	0.167	0.336	0.620	1.181	0.611	2.283			
		Inverse variance weighted	17	0.080	0.285	0.779	1.083	0.620	1.892	0.598		
		Simple mode	17	-0.225	0.751	0.769	0.799	0.183	3.480			
		Weighted mode	17	0.199	0.340	0.566	1.220	0.627	2.374			
		MR-PRESSO	17	0.152	0.124	0.229	1.164	0.913	1.485			
**finn-a-I9_MI**	Myocardial infarction	MR Egger	17	0.192	0.314	0.547	1.211	0.654	2.243	0.063	0.938	0.098
		Weighted median	17	0.262	0.198	0.186	1.299	0.882	1.915			
		Inverse variance weighted	17	0.170	0.158	0.281	1.186	0.870	1.617	0.080		
		Simple mode	17	0.223	0.388	0.570	1.250	0.585	2.671			
		Weighted mode	17	0.276	0.191	0.158	1.318	0.906	1.917			

		MR-PRESSO	17	0.220	0.128	0.096	1.246	0.969	1.602			
**finn-a-I9_ICH**	Intracranial haemorrhage	MR Egger	17	3.099	0.959	**0.006**	22.17	3.386	145.2	0.249	0.842	0.105
		Weighted median	17	2.308	0.690	**0.001**	10.04	2.600	38.83			
		Inverse variance weighted	17	1.131	0.563	**0.045**	3.099	1.028	9.340	0.289		
		Simple mode	17	-0.001	1.679	1.000	0.999	0.037	26.82			
		Weighted mode	17	2.285	0.727	**0.006**	9.827	2.364	40.84			
		MR-PRESSO	17	-0.068	0.217	0.757	0.934	0.610	1.430			
**finn-a-HEARTFAIL**	Heart failure	MR Egger	17	-0.415	0.282	0.152	0.660	0.380	1.147	0.101	0.519	0.058
		Weighted median	17	-0.282	0.198	0.155	0.755	0.512	1.113			
		Inverse variance weighted	17	-0.257	0.143	0.072	0.774	0.585	1.023	0.113		
		Simple mode	17	0.521	0.416	0.219	1.685	0.746	3.805			
		Weighted mode	17	-0.244	0.209	0.252	0.783	0.520	1.180			
		MR-PRESSO	17	-0.110	0.120	0.362	0.895	0.708	1.132			
**finn-a-I9_ISCHHEART**	Ischemic heart disease	MR Egger	17	-0.107	0.209	0.612	0.898	0.596	1.354	0.166	0.295	0.077
		Weighted median	17	0.047	0.145	0.743	1.048	0.790	1.392			
		Inverse variance weighted	17	0.085	0.107	0.429	1.088	0.882	1.343	0.157		
		Simple mode	17	0.087	0.261	0.740	1.091	0.654	1.822			
		Weighted mode	17	0.054	0.138	0.699	1.055	0.805	1.383			
		MR-PRESSO	17	0.147	0.093	0.120	1.159	0.967	1.389			
**finn-a-I9_CORATHER**	Coronary atherosclerosis	MR Egger	17	0.188	0.229	0.418	1.207	0.771	1.890	0.244	0.904	0.231
		Weighted median	17	0.224	0.169	0.186	1.251	0.898	1.743			

		Inverse variance weighted	17	0.164	0.115	0.154	1.178	0.940	1.477	0.286			
		Simple mode	17	0.446	0.318	0.171	1.563	0.837	2.917				
		Weighted mode	17	0.275	0.167	0.110	1.316	0.949	1.827				
		MR-PRESSO	17	0.224	0.097	**0.027**	1.250	1.034	1.512				
**finn-a-I9_CHD**	Major coronary heart disease event	MR Egger	17	0.098	0.265	0.715	1.103	0.656	1.854	0.048	0.655	0.022	NA*
		Weighted median	17	0.147	0.164	0.368	1.159	0.841	1.597				
		Inverse variance weighted	17	-0.005	0.134	0.969	0.995	0.765	1.293	0.057			
		Simple mode	17	-0.180	0.296	0.546	0.835	0.468	1.490				
		Weighted mode	17	0.084	0.154	0.590	1.087	0.804	1.471				
		MR-PRESSO	17	0.121	0.113	0.294	1.128	0.903	1.409				
**finn-a-I9_PAD**	Peripheral artery disease	MR Egger	17	0.405	0.527	0.449	1.499	0.533	4.213	0.000	0.801	<0.001	NA*
		Weighted median	17	0.325	0.244	0.183	1.383	0.858	2.231				
		Inverse variance weighted	17	0.520	0.265	**0.050**	1.682	1.000	2.831	0.000			
		Simple mode	17	0.819	0.461	0.086	2.267	0.918	5.599				
		Weighted mode	17	0.380	0.230	0.108	1.462	0.932	2.293				
		MR-PRESSO	17	0.441	0.212	**0.045**	1.555	1.027	2.353				
**finn-a-I9_HYPTENSESS**	Essential hypertension	MR Egger	17	-0.500	0.212	**0.025**	0.606	0.400	0.918	0.002	0.081	<0.001	0.143
		Weighted median	17	-0.251	0.113	**0.026**	0.778	0.623	0.971				
		Inverse variance weighted	17	-0.172	0.113	0.127	0.842	0.675	1.050	0.001			
		Simple mode	17	-0.180	0.195	0.361	0.835	0.570	1.223				
		Weighted mode	17	-0.279	0.101	**0.009**	0.756	0.621	0.921				

		MR-PRESSO	17	-0.085	0.095	0.382	0.919	0.762	1.108				
**finn-a-C_STROKE**	Stroke	MR Egger	17	-0.329	0.224	0.153	0.720	0.464	1.116	0.300	0.029	0.149	
		Weighted median	17	0.051	0.160	0.750	1.052	0.769	1.441				
		Inverse variance weighted	17	0.112	0.122	0.359	1.119	0.880	1.422	0.141			
		Simple mode	17	0.106	0.304	0.729	1.112	0.613	2.018				
		Weighted mode	17	-0.099	0.165	0.551	0.905	0.656	1.251				
		MR-PRESSO	17	0.128	0.097	0.198	1.136	0.939	1.375				
**finn-a-I9_STR_EXH**	Ischemic stroke	MR Egger	17	0.044	0.294	0.882	1.045	0.587	1.860	0.102	0.866	0.165	
		Weighted median	17	-0.074	0.196	0.708	0.929	0.632	1.365				
		Inverse variance weighted	17	0.087	0.148	0.557	1.091	0.816	1.459	0.126			
		Simple mode	17	0.300	0.382	0.439	1.349	0.638	2.854				
		Weighted mode	17	-0.019	0.204	0.925	0.981	0.658	1.463				
		MR-PRESSO	17	0.124	0.120	0.308	1.133	0.895	1.433				
**finn-a-I9_AF**	Atrial fibrillation and fluttering	MR Egger	17	-0.164	0.293	0.579	0.848	0.478	1.506	0.089	0.719	0.014	NA*
		Weighted median	17	-0.216	0.194	0.266	0.806	0.552	1.178				
		Inverse variance weighted	17	-0.256	0.148	0.083	0.774	0.580	1.034	0.107			
		Simple mode	17	-0.013	0.352	0.971	0.987	0.495	1.967				
		Weighted mode	17	-0.222	0.204	0.286	0.801	0.537	1.195				
		MR-PRESSO	17	-0.057	0.132	0.669	0.945	0.730	1.223				
**The Global Lipids Genetics Consortium**													
**ieu-a-299**	HDL cholesterol	MR Egger	35	0.030	0.153	0.845	1.030	0.764	1.390	0.000	0.716	<0.001	0.915

		Weighted median	35	0.040	0.037	0.285	1.040	0.968	1.119				
		Inverse variance weighted	35	0.077	0.083	0.357	1.080	0.917	1.271	0.000			
		Simple mode	35	0.040	0.072	0.583	1.041	0.903	1.199				
		Weighted mode	35	0.040	0.033	0.238	1.041	0.975	1.111				
		MR-PRESSO	35	0.077	0.083	0.363	1.080	0.917	1.271				
**ieu-a-300**	LDL cholesterol	MR Egger	35	-0.070	0.169	0.680	0.932	0.670	1.297	0.000	0.423	<0.001	0.657
		Weighted median	35	0.017	0.043	0.685	1.018	0.935	1.107				
		Inverse variance weighted	35	0.044	0.092	0.635	1.045	0.872	1.253	0.000			
		Simple mode	35	0.027	0.091	0.765	1.028	0.860	1.228				
		Weighted mode	35	0.027	0.040	0.503	1.028	0.950	1.112				
		MR-PRESSO	35	0.044	0.092	0.638	1.045	0.872	1.253				
**ieu-a-302**	Triglycerides	MR Egger	35	-0.070	0.281	0.804	0.932	0.538	1.616	0.000	0.671	<0.001	<0.001
		Weighted median	35	0.023	0.036	0.536	1.023	0.952	1.099				
		Inverse variance weighted	35	-0.171	0.153	0.263	0.843	0.625	1.137	0.000			
		Simple mode	35	-0.004	0.065	0.946	0.996	0.876	1.131				
		Weighted mode	35	0.020	0.029	0.501	1.020	0.963	1.080				
		MR-PRESSO	35	-0.171	0.153	0.271	0.843	0.625	1.137				

*No significant outliers

Table S6 The causal effect of genetically predicted FI on outcomes in different studies.

ID of outcome	Outcome	Methods	No. of SNPs	Beta	SE	*p* value	OR	LCI	UCI	Heterogeneity *p* value	*p* value for intercept	*p* value for MR-PRESSO	Distortion test *p* value
**ukb-d-I9_K_CARDIAC**	Cardiovascular mortality	MR Egger	18	0.018	0.016	0.279	1.018	0.987	1.050	0.415	0.376	0.434	
		Weighted median	18	0.006	0.004	0.144	1.006	0.998	1.014				
		Inverse variance weighted	18	0.004	0.003	0.216	1.004	0.998	1.009	0.428			
		Simple mode	18	0.007	0.007	0.311	1.007	0.994	1.020				
		Weighted mode	18	0.005	0.006	0.417	1.005	0.993	1.018				
		MR-PRESSO	18	0.004	0.003	0.238	1.004	0.998	1.009				
**ieu-a-798**	Myocardial infarction	MR Egger	17	-1.531	1.390	0.292	0.216	0.014	3.297	0.005	0.251	0.006	NA*
		Weighted median	17	0.529	0.295	0.073	1.697	0.953	3.024				
		Inverse variance weighted	17	0.596	0.271	**0.028**	1.815	1.068	3.085	0.003			
		Simple mode	17	0.358	0.587	0.552	1.431	0.453	4.525				
		Weighted mode	17	0.300	0.508	0.565	1.350	0.499	3.654				
		MR-PRESSO	17	0.501	0.243	0.055	1.650	1.025	2.655				
**ukb-d-I9_INTRACRA**	Intracranial haemorrhage	MR Egger	17	-0.006	0.019	0.763	0.994	0.959	1.031	0.016	0.824	0.022	0.507
		Weighted median	17	-0.005	0.003	0.117	0.995	0.989	1.001				
		Inverse variance weighted	17	-0.002	0.003	0.597	0.998	0.993	1.004	0.023			
		Simple mode	17	-0.005	0.005	0.315	0.995	0.985	1.005				
		Weighted mode	17	-0.005	0.005	0.258	0.995	0.986	1.004				
		MR-PRESSO	17	-0.004	0.002	0.110	0.996	0.991	1.001				

**ebi-a-GCST009541**	Heart failure	MR Egger	18	1.548	1.254	0.235	4.702	0.402	54.92	0.000	0.285	<0.001	
		Weighted median	18	-0.036	0.182	0.842	0.964	0.675	1.379				
		Inverse variance weighted	18	0.179	0.205	0.384	1.196	0.800	1.788	0.000			
		Simple mode	18	-0.012	0.293	0.968	0.988	0.556	1.755				
		Weighted mode	18	-0.040	0.279	0.889	0.961	0.557	1.660				
		MR-PRESSO	18	0.179	0.205	0.396	1.196	0.800	1.788				
**ieu-a-7**	Ischemic heart disease	MR Egger	18	-1.180	1.452	0.428	0.307	0.018	5.294	0.001	0.203	0.002	0.459
		Weighted median	18	0.815	0.246	**0.001**	2.260	1.397	3.657				
		Inverse variance weighted	18	0.721	0.243	**0.003**	2.057	1.278	3.311	0.000			
		Simple mode	18	1.291	0.487	**0.017**	3.636	1.400	9.441				
		Weighted mode	18	1.291	0.503	**0.020**	3.636	1.358	9.738				
		MR-PRESSO	18	0.721	0.243	**0.009**	2.057	1.278	3.311				
**ukb-d-I9_CORATHER**	Coronary atherosclerosis	MR Egger	18	-0.144	0.061	**0.035**	0.866	0.769	0.975	0.037	0.012	<0.001	0.484
		Weighted median	18	0.044	0.014	**0.001**	1.045	1.017	1.074				
		Inverse variance weighted	18	0.032	0.014	**0.024**	1.033	1.004	1.062	0.000			
		Simple mode	18	0.060	0.026	**0.037**	1.062	1.009	1.116				
		Weighted mode	18	0.058	0.023	**0.026**	1.060	1.013	1.108				
		MR-PRESSO	18	0.040	0.011	**0.005**	1.041	1.018	1.064				
**ukb-d-I9_CHD**	Major coronary heart disease event	MR Egger	18	-0.100	0.044	**0.041**	0.905	0.830	0.985	0.209	0.015	0.016	NA*
		Weighted median	18	0.024	0.011	**0.032**	1.024	1.002	1.047				
		Inverse varianc	18	0.021	0.010	**0.039**	1.021	1.001	1.041	0.017			

		e weighted											
		Simple mode	18	0.029	0.022	0.220	1.029	0.985	1.076				
		Weighted mode	18	0.023	0.021	0.289	1.024	0.982	1.067				
		MR-PRESSO	18	0.021	0.010	0.060	1.021	1.001	1.041				
**bbj-a-144**	Peripheral artery disease	MR Egger	17	1.121	3.647	0.763	3.068	0.002	3901.3	0.094	0.964	0.141	
		Weighted median	17	1.969	0.641	**0.002**	7.163	2.039	25.16				
		Inverse variance weighted	17	1.285	0.445	**0.004**	3.614	1.510	8.650	0.126			
		Simple mode	17	2.223	0.916	**0.027**	9.236	1.535	55.56				
		Weighted mode	17	2.160	0.913	**0.031**	8.675	1.449	51.93				
		MR-PRESSO	17	1.285	0.529	**0.027**	3.614	1.282	10.18				
**ukb-b-12493**	Essential hypertension	MR Egger	18	0.087	0.115	0.467	1.091	0.870	1.367	0.000	0.984	<0.001	0.326
		Weighted median	18	0.095	0.021	**0.000**	1.100	1.056	1.146				
		Inverse variance weighted	18	0.089	0.021	**0.000**	1.093	1.050	1.138	0.000			
		Simple mode	18	0.138	0.037	**0.003**	1.148	1.067	1.235				
		Weighted mode	18	0.136	0.035	**0.002**	1.146	1.069	1.228				
		MR-PRESSO	18	0.111	0.018	**0.000**	1.117	1.078	1.158				
**ebi-a-GCST006906**	Stroke	MR Egger	17	0.121	1.106	0.915	1.128	0.129	9.868	0.084	0.931	0.123	
		Weighted median	17	0.173	0.218	0.430	1.188	0.774	1.823				
		Inverse variance weighted	17	0.196	0.188	0.298	1.216	0.841	1.759	0.113			
		Simple mode	17	0.344	0.346	0.336	1.410	0.715	2.781				
		Weighted mode	17	0.235	0.355	0.517	1.265	0.631	2.537				
		MR-PRESSO	17	0.024	0.182	0.897	1.024	0.717	1.464				

**ebi-a-GCST006908**	Ischemic stroke	MR Egger	17	-0.151	1.166	0.898	0.859	0.087	8.445	0.356		0.945	0.155	
		Weighted median	17	0.257	0.220	0.242	1.293	0.840	1.991					
		Inverse variance weighted	17	0.147	0.187	0.431	1.158	0.803	1.670	0.434				
		Simple mode	17	0.476	0.361	0.207	1.609	0.792	3.267					
		Weighted mode	17	0.355	0.368	0.349	1.426	0.693	2.935					
		MR-PRESSO	17	0.017	0.191	0.931	1.017	0.699	1.479					
**ebi-a-GCST006414**	Atrial fibrillation and fluttering	MR Egger	17	0.337	1.135	0.771	1.401	0.151	12.95	0.000		0.720	<0.001	0.547
		Weighted median	17	-0.143	0.174	0.412	0.867	0.617	1.219					
		Inverse variance weighted	17	-0.071	0.185	0.701	0.931	0.648	1.339	0.000				
		Simple mode	17	-0.334	0.301	0.283	0.716	0.397	1.291					
		Weighted mode	17	-0.294	0.317	0.367	0.745	0.400	1.387					
		MR-PRESSO	17	-0.071	0.185	0.706	0.931	0.648	1.339					
**ukb-d-30760_irnt**	HDL cholesterol	MR Egger	18	-0.290	0.557	0.610	0.748	0.251	2.231	0.000		0.913	<0.001	0.746
		Weighted median	18	-0.201	0.038	**0.000**	0.818	0.759	0.881					
		Inverse variance weighted	18	-0.351	0.088	**0.000**	0.704	0.593	0.837	0.000				
		Simple mode	18	-0.376	0.075	**0.000**	0.687	0.593	0.795					
		Weighted mode	18	-0.183	0.095	0.070	0.833	0.692	1.002					
		MR-PRESSO	18	-0.351	0.088	**0.001**	0.704	0.593	0.837					
**ukb-d-30780_irnt**	LDL cholesterol	MR Egger	18	-2.295	0.892	**0.020**	0.101	0.018	0.578	0.000		0.030	<0.001	1

		Weighted median	18	-0.005	0.072	0.943	0.995	0.864	1.146	0.000			
		Inverse variance weighted	18	-0.204	0.164	0.213	0.816	0.592	1.124				
		Simple mode	18	0.044	0.123	0.724	1.045	0.821	1.331				
		Weighted mode	18	-0.003	0.092	0.977	0.997	0.832	1.195				
		MR-PRESSO	18	-0.204	0.164	0.230	0.816	0.592	1.124				
**ukb-d-30870_irnt**	Triglycerides	MR Egger	18	-3.583	2.768	0.214	0.028	0.000	6.314	0.000	0.176	<0.001	1
		Weighted median	18	0.209	0.091	**0.022**	1.232	1.031	1.473				
		Inverse variance weighted	18	0.283	0.464	0.541	1.328	0.535	3.294	0.000			
		Simple mode	18	0.335	0.252	0.201	1.398	0.853	2.290				
		Weighted mode	18	0.064	0.065	0.343	1.066	0.938	1.211				
		MR-PRESSO	18	0.283	0.464	0.549	1.328	0.535	3.294				
**ukb-d-30630_irnt**	Apolipoprotein A	MR Egger	18	-0.387	0.327	0.254	0.679	0.358	1.288	0.000	0.646	<0.001	0.343
		Weighted median	18	-0.109	0.028	**0.000**	0.896	0.849	0.947				
		Inverse variance weighted	18	-0.236	0.052	**0.000**	0.790	0.713	0.874	0.000			
		Simple mode	18	-0.294	0.051	**0.000**	0.746	0.674	0.824				
		Weighted mode	18	-0.065	0.039	0.111	0.937	0.868	1.011				
		MR-PRESSO	18	-0.236	0.052	**0.000**	0.790	0.713	0.874				
**ukb-d-30640_irnt**	Apolipoprotein B	MR Egger	18	-0.638	0.317	**0.041**	0.528	0.284	0.983	0.000	0.068	<0.001	<0.001
		Weighted median	18	0.037	0.022	0.097	1.038	0.993	1.085				
		Inverse variance	18	-0.025	0.056	0.655	0.975	0.874	1.088	0.000			

		weighted											
		Simple mode	18	0.093	0.030	**0.006**	1.098	1.035	1.163				
		Weighted mode	18	0.080	0.030	**0.017**	1.083	1.021	1.149				
		MR-PRESSO	18	-0.025	0.056	0.661	0.975	0.874	1.088				
**ukb-d-30790_irnt**	Lipoprotein A	MR Egger	18	0.141	0.360	0.701	1.151	0.569	2.331	0.017	0.448	0.017	NA*
		Weighted median	18	-0.081	0.066	0.224	0.923	0.810	1.051				
		Inverse variance weighted	18	-0.135	0.058	**0.019**	0.873	0.780	0.978	0.018			
		Simple mode	18	-0.085	0.144	0.565	0.919	0.692	1.219				
		Weighted mode	18	-0.067	0.123	0.593	0.935	0.734	1.191				
		MR-PRESSO	18	-0.135	0.058	**0.032**	0.873	0.780	0.978				
**FinnGen Study**													
**finn-a-I9_K_CARDIAC**	Cardiovascular mortality	MR Egger	18	1.572	1.775	0.389	4.817	0.149	156.1	0.384	0.498	0.420	
		Weighted median	18	0.520	0.396	0.189	1.682	0.774	3.656				
		Inverse variance weighted	18	0.359	0.294	0.221	1.432	0.806	2.546	0.419			
		Simple mode	18	0.546	0.828	0.518	1.727	0.341	8.742				
		Weighted mode	18	0.744	0.730	0.322	2.104	0.503	8.793				
		MR-PRESSO	18	0.359	0.294	0.238	1.432	0.806	2.546				
**finn-a-I9_MI**	Myocardial infarction	MR Egger	18	-1.220	2.118	0.573	0.295	0.005	18.73	0.005	0.331	0.003	0.111
		Weighted median	18	0.703	0.371	0.058	2.019	0.976	4.174				
		Inverse variance weighted	18	0.873	0.356	**0.014**	2.393	1.192	4.805	0.004			
		Simple mode	18	0.557	0.727	0.454	1.746	0.420	7.262				

		Weighted mode	18	0.576	0.728	0.440	1.779	0.427	7.411				
		MR-PRESSO	18	0.873	0.356	**0.025**	2.393	1.192	4.805				
**finn-a-I9_ICH**	Intracranial haemorrhage	MR Egger	18	-4.098	2.553	0.128	0.017	0.000	2.473	0.726	0.096	0.596	
		Weighted median	18	0.867	0.584	0.138	2.380	0.757	7.480				
		Inverse variance weighted	18	0.354	0.428	0.408	1.425	0.616	3.300	0.567			
		Simple mode	18	0.858	1.025	0.414	2.359	0.317	17.57				
		Weighted mode	18	0.878	0.999	0.392	2.406	0.339	100.6				
		MR-PRESSO	18	0.354	0.408	0.397	1.425	0.641	3.168				
**finn-a-HEARTFAIL**	Heart failure	MR Egger	18	1.518	1.579	0.351	4.563	0.207	100.6	0.131	0.591	0.167	
		Weighted median	18	0.574	0.338	0.089	1.775	0.916	3.441				
		Inverse variance weighted	18	0.665	0.259	**0.010**	1.945	1.170	3.234	0.155			
		Simple mode	18	0.846	0.691	0.237	2.330	0.602	9.018				
		Weighted mode	18	0.526	0.726	0.479	1.692	0.407	7.026				
		MR-PRESSO	18	0.665	0.259	**0.020**	1.945	1.170	3.234				
**finn-a-I9_ISCHHEART**	Ischemic heart disease	MR Egger	18	-1.985	1.948	0.324	0.137	0.003	6.261	0.000	0.186	<0.001	0.438
		Weighted median	18	0.577	0.294	**0.049**	1.781	1.002	3.167				
		Inverse variance weighted	18	0.669	0.336	**0.046**	1.952	1.011	3.771	0.000			
		Simple mode	18	0.984	0.733	0.197	2.674	0.636	11.25				
		Weighted mode	18	1.018	0.756	0.196	2.767	0.629	12.17				
		MR-PRESSO	18	0.669	0.336	0.063	1.952	1.011	3.771				
**finn-a-I9_CORATHER**	Coronary atherosclerosis	MR Egger	18	-1.342	1.972	0.506	0.261	0.005	12.46	0.000	0.299	<0.001	0.305

		Weighted median	18	0.783	0.329	**0.017**	2.188	1.149	4.170				
		Inverse variance weighted	18	0.745	0.332	**0.025**	2.106	1.097	4.040	0.000			
		Simple mode	18	1.015	0.680	0.154	2.760	0.728	10.45				
		Weighted mode	18	1.120	0.641	0.099	3.066	0.873	10.76				
		MR-PRESSO	18	0.745	0.332	**0.039**	2.106	1.097	4.040				
**finn-a-I9_CHD**	Major coronary heart disease event	MR Egger	18	-2.990	1.935	0.142	0.050	0.001	2.233	0.000	0.085	<0.001	0.244
		Weighted median	18	0.608	0.337	0.071	1.837	0.949	3.554				
		Inverse variance weighted	18	0.517	0.347	0.137	1.676	0.849	3.309	0.000			
		Simple mode	18	1.261	0.722	0.099	3.527	0.857	14.52				
		Weighted mode	18	1.104	0.778	0.174	3.015	0.657	13.84				
		MR-PRESSO	18	0.517	0.347	0.155	1.676	0.849	3.309				
**finn-a-I9_PAD**	Peripheral artery disease	MR Egger	18	-1.732	2.180	0.438	0.177	0.002	12.67	0.052	0.246	0.048	NA*
		Weighted median	18	1.079	0.420	**0.010**	2.941	1.292	6.697				
		Inverse variance weighted	18	0.855	0.371	**0.021**	2.351	1.136	4.865	0.039			
		Simple mode	18	1.024	0.630	0.123	2.784	0.809	9.578				
		Weighted mode	18	1.044	0.588	0.094	2.840	0.897	8.987				
		MR-PRESSO	18	0.855	0.371	**0.034**	2.351	1.136	4.865				
**finn-a-I9_HYPTENSESS**	Essential hypertension	MR Egger	18	-0.101	1.806	0.956	0.904	0.026	31.15	0.000	0.556	<0.001	0.364
		Weighted median	18	1.363	0.259	**0.000**	3.908	2.351	6.498				
		Inverse variance weighted	18	0.969	0.297	**0.001**	2.634	1.471	4.719	0.000			

		Simple mode	18	1.604	0.370	**0.000**	4.973	2.410	10.26				
		Weighted mode	18	1.604	0.493	**0.005**	4.973	1.891	13.07				
		MR-PRESSO	18	0.969	0.297	**0.005**	2.634	1.471	4.719				
**finn-a-C_STROKE**	Stroke	MR Egger	18	-2.539	1.282	0.065	0.079	0.006	0.973	0.244	0.024	0.075	
		Weighted median	18	0.384	0.282	0.174	1.467	0.844	2.552				
		Inverse variance weighted	18	0.617	0.246	**0.012**	1.853	1.144	3.004	0.057			
		Simple mode	18	0.284	0.506	0.582	1.329	0.493	3.583				
		Weighted mode	18	0.238	0.492	0.635	1.268	0.484	3.326				
		MR-PRESSO	18	0.617	0.246	**0.023**	1.853	1.144	3.004				
**finn-a-I9_STR_EXH**	Ischemic stroke	MR Egger	18	0.136	1.396	0.923	1.146	0.074	17.67	0.645	0.648	0.703	
		Weighted median	18	0.791	0.321	**0.014**	2.205	1.176	4.134				
		Inverse variance weighted	18	0.777	0.234	**0.001**	2.175	1.374	3.441	0.696			
		Simple mode	18	0.970	0.567	0.105	2.638	0.868	8.016				
		Weighted mode	18	0.864	0.525	0.118	2.373	0.848	6.640				
		MR-PRESSO	18	0.777	0.209	**0.002**	2.175	1.443	3.278				
**finn-a-I9_AF**	Atrial fibrillation and fluttering	MR Egger	18	1.938	1.853	0.311	6.944	0.184	262.3	0.023	0.495	0.027	NA*
		Weighted median	18	0.624	0.359	0.082	1.866	0.923	3.774				
		Inverse variance weighted	18										
		Simple mode	18	0.636	0.677	0.361	1.888	0.501	7.114				
		Weighted mode	18	0.685	0.645	0.303	1.984	0.560	7.022				
		MR-PRESSO	18	0.661	0.306	**0.046**	1.937	1.062	3.531				
**The Global Lipids**													

**Genetics Consortium**													
**ieu-a-299**	HDL cholesterol	MR Egger	18	-0.587	1.335	0.666	0.556	0.041	7.603	0.000	0.772	<0.001	0.513
		Weighted median	18	-0.873	0.124	**0.000**	0.418	0.328	0.532				
		Inverse variance weighted	18	-0.976	0.216	**0.000**	0.377	0.247	0.575	0.000			
		Simple mode	18	-1.099	0.234	**0.000**	0.333	0.210	0.528				
		Weighted mode	18	-0.829	0.239	**0.003**	0.437	0.274	0.697				
		MR-PRESSO	18	-0.976	0.216	**0.000**	0.377	0.247	0.575				
**ieu-a-300**	LDL cholesterol	MR Egger	18	-2.786	1.226	**0.037**	0.062	0.006	0.681	0.000	0.046	<0.001	0.001
		Weighted median	18	-0.002	0.113	0.986	0.998	0.799	1.246				
		Inverse variance weighted	18	-0.172	0.224	0.441	0.842	0.543	1.305	0.000			
		Simple mode	18	0.127	0.172	0.469	1.136	0.811	1.591				
		Weighted mode	18	0.090	0.131	0.501	1.094	0.847	1.414				
		MR-PRESSO	18	-0.172	0.224	0.452	0.842	0.543	1.305				
**ieu-a-302**	Triglycerides	MR Egger	18	-2.711	2.858	0.357	0.066	0.000	18.01	0.000	0.306	<0.001	1
		Weighted median	18	0.665	0.111	**0.000**	1.944	1.566	2.415				
		Inverse variance weighted	18	0.271	0.472	0.566	1.311	0.519	3.308	0.000			
		Simple mode	18	0.691	0.273	**0.022**	1.995	1.168	3.407				
		Weighted mode	18	0.520	0.210	**0.024**	1.681	1.115	2.536				
		MR-PRESSO	18	0.271	0.472	0.574	1.311	0.519	3.308				

*No significant outliers

Table S7 The causal effect of genetically predicted HbA_{1c} on outcomes in different studies.

ID of outcome	Outcome	Methods	No. of SNPs	Beta	SE	*p* value	OR	LCI	UCI	Heterogeneity *p* value	*p* value for intercept	*p* value for MR-PRESSO	Distortion test *p* value
**ukb-d-I9_K_CARDIAC**	Cardiovascular mortality	MR Egger	34	-0.001	0.003	0.723	0.999	0.993	1.005	0.792	0.162	0.504	
		Weighted median	34	0.001	0.002	0.560	1.001	0.997	1.006				
		Inverse variance weighted	34	0.002	0.002	0.124	1.002	0.999	1.006	0.743			
		Simple mode	34	0.005	0.004	0.228	1.005	0.997	1.014				
		Weighted mode	34	0.000	0.002	0.973	1.000	0.995	1.005				
		MR-PRESSO	34	0.002	0.002	0.130	1.002	0.999	1.005				
**ieu-a-798**	Myocardial infarction	MR Egger	34	0.531	0.511	0.306	1.701	0.625	4.627	0.000	0.520	<0.001	0.537
		Weighted median	34	0.389	0.169	**0.021**	1.475	1.059	2.055				
		Inverse variance weighted	34	0.222	0.110	**0.043**	1.249	1.007	1.550	0.000			
		Simple mode	34	0.271	0.279	0.338	1.311	0.759	2.266				
		Weighted mode	34	0.302	0.189	0.119	1.353	0.934	1.959				
		MR-PRESSO	34	0.249	0.230	0.287	1.282	0.817	2.014				
**ukb-d-I9_INTRACRA**	Intracranial haemorrhage	MR Egger	34	-0.001	0.003	0.604	0.999	0.994	1.004	0.783	0.885	0.811	
		Weighted median	34	0.000	0.002	0.910	1.000	0.996	1.004				
		Inverse variance weighted	34	-0.001	0.001	0.464	0.999	0.996	1.002	0.818			
		Simple mode	34	-0.003	0.003	0.310	0.997	0.990	1.003				
		Weighted mode	34	-0.001	0.002	0.696	0.999	0.995	1.003				
		MR-PRESSO	34	-0.001	0.001	0.485	0.999	0.997	1.002				
**ebi-a-GCST009541**	Heart failure	MR Egger	31	0.266	0.251	0.299	1.304	0.797	2.134	0.000	0.075	<0.001	0.839

		Weighted median	31	0.002	0.133	0.988	1.002	0.772	1.301				
		Inverse variance weighted	31	-0.120	0.145	0.408	0.887	0.667	1.179	0.000			
		Simple mode	31	-0.263	0.202	0.203	0.768	0.517	1.142				
		Weighted mode	31	-0.017	0.130	0.898	0.983	0.763	1.268				
		MR-PRESSO	31	-0.120	0.141	0.400	0.887	0.673	1.169				
**ieu-a-7**	Ischemic heart disease	MR Egger	34	0.533	0.241	0.055	1.704	1.062	2.733	0.625	0.286	<0.001	0.309
		Weighted median	34	0.319	0.127	**0.012**	1.376	1.072	1.765				
		Inverse variance weighted	34	0.283	0.099	**0.004**	1.327	1.094	1.611	0.590			
		Simple mode	34	0.294	0.196	0.165	1.342	0.913	1.973				
		Weighted mode	34	0.322	0.152	0.061	1.380	1.024	1.860				
		MR-PRESSO	34	0.353	0.195	0.079	1.423	0.970	2.087				
**ukb-d-I9_CORATHER**	Coronary atherosclerosis	MR Egger	34	0.004	0.013	0.744	1.004	0.980	1.030	0.000	0.467	0.102	
		Weighted median	34	0.011	0.006	0.063	1.011	0.999	1.024				
		Inverse variance weighted	34	0.019	0.006	**0.001**	1.019	1.008	1.031	0.000			
		Simple mode	34	0.015	0.011	0.205	1.015	0.993	1.038				
		Weighted mode	34	0.012	0.006	0.068	1.012	1.000	1.025				
		MR-PRESSO	34	0.015	0.006	**0.018**	1.015	1.003	1.026				
**ukb-d-I9_CHD**	Major coronary heart disease event	MR Egger	34	-0.007	0.011	0.513	0.993	0.971	1.015	0.000	0.955	<0.001	0.305
		Weighted median	34	-0.012	0.006	0.059	0.988	0.976	1.000				
		Inverse variance weighted	34	-0.007	0.006	0.255	0.993	0.981	1.005	0.000			
		Simple mode	34	0.022	0.013	0.084	1.023	0.998	1.048				

		Weighted mode	34	-0.013	0.007	0.068	0.987	0.973	1.001				
		MR-PRESSO	34	-0.001	0.005	0.805	0.999	0.989	1.008				
**bbj-a-144**	Peripheral artery disease	MR Egger	28	0.911	1.184	0.449	2.486	0.244	25.32	0.027	0.522	0.021	NA*
		Weighted median	28	0.776	0.556	0.163	2.172	0.731	6.455				
		Inverse variance weighted	28	0.200	0.447	0.654	1.222	0.508	2.937	0.031			
		Simple mode	28	1.803	0.940	0.066	6.066	0.961	38.30				
		Weighted mode	28	1.020	0.618	0.110	2.773	0.826	9.310				
		MR-PRESSO	28	0.231	0.393	0.561	1.260	0.583	2.722				
**ukb-b-12493**	Essential hypertension	MR Egger	34	0.013	0.034	0.700	1.013	0.948	1.083	0.000	0.686	<0.001	0.562
		Weighted median	34	0.030	0.010	**0.005**	1.030	1.009	1.051				
		Inverse variance weighted	34	0.002	0.018	0.929	1.002	0.966	1.038	0.000			
		Simple mode	34	0.027	0.015	0.082	1.027	0.998	1.057				
		Weighted mode	34	0.027	0.010	**0.012**	1.027	1.007	1.047				
		MR-PRESSO	34	0.022	0.009	**0.023**	1.022	1.004	1.041				
**ebi-a-GCST006906**	Stroke	MR Egger	33	-0.173	0.302	0.571	0.841	0.465	1.521	0.000	0.920	<0.001	0.304
		Weighted median	33	-0.043	0.145	0.769	0.958	0.721	1.273				
		Inverse variance weighted	33	-0.148	0.162	0.362	0.863	0.628	1.185	0.000			
		Simple mode	33	-0.187	0.276	0.503	0.829	0.482	1.426				
		Weighted mode	33	-0.120	0.159	0.456	0.887	0.649	1.211				
		MR-PRESSO	33	-0.088	0.151	0.562	0.916	0.682	1.230				
**ebi-a-GCST006908**	Ischemic stroke	MR Egger	33	-0.141	0.345	0.686	0.869	0.442	1.708	0.000	0.946	<0.001	0.259

		Weighted median	33	-0.052	0.167	0.756	0.949	0.684	1.318				
		Inverse variance weighted	33	-0.161	0.185	0.384	0.851	0.593	1.223	0.000			
		Simple mode	33	-0.031	0.287	0.915	0.970	0.552	1.702				
		Weighted mode	33	-0.107	0.172	0.539	0.899	0.642	1.258				
		MR-PRESSO	33	-0.092	0.172	0.597	0.912	0.651	1.278				
**ebi-a-GCST006414**	Atrial fibrillation and fluttering	MR Egger	33	-0.043	0.176	0.810	0.958	0.679	1.352	0.002	0.883	0.008	0.733
		Weighted median	33	-0.096	0.105	0.358	0.908	0.739	1.115				
		Inverse variance weighted	33	-0.064	0.093	0.490	0.938	0.781	1.126	0.003			
		Simple mode	33	-0.148	0.177	0.410	0.862	0.609	1.221				
		Weighted mode	33	-0.102	0.115	0.384	0.903	0.721	1.132				
		MR-PRESSO	33	-0.091	0.085	0.295	0.913	0.773	1.080				
**ukb-d-30760_irnt**	HDL cholesterol	MR Egger	34	0.070	0.060	0.252	1.072	0.953	1.206	0.000	0.204	<0.001	0.894
		Weighted median	34	0.023	0.015	0.114	1.024	0.994	1.054				
		Inverse variance weighted	34	0.005	0.033	0.885	1.005	0.941	1.072	0.000			
		Simple mode	34	0.033	0.023	0.154	1.034	0.989	1.081				
		Weighted mode	34	0.025	0.014	0.081	1.025	0.998	1.053				
		MR-PRESSO	34	0.000	0.030	0.997	1.000	0.943	1.061				
**ukb-d-30780_irnt**	LDL cholesterol	MR Egger	34	0.307	0.184	0.104	1.360	0.949	1.949	0.000	0.436	<0.001	<0.001
		Weighted median	34	0.171	0.037	**0.000**	1.186	1.102	1.276				
		Inverse variance weighted	34	0.186	0.021	**0.000**	1.205	1.157	1.255	0.000			
		Simple mode	34	0.048	0.072	0.514	1.049	0.910	1.209				

		Weighted mode	34	0.140	0.034	**0.000**	1.150	1.076	1.230				
		MR-PRESSO	34	0.169	0.095	0.085	1.184	0.982	1.426				
**ukb-d-30870_irnt**	Triglycerides	MR Egger	34	-0.129	0.126	0.315	0.879	0.686	1.126	0.000	0.069	<0.001	<0.001
		Weighted median	34	-0.023	0.039	0.564	0.978	0.906	1.056				
		Inverse variance weighted	34	0.070	0.072	0.329	1.073	0.932	1.235	0.000			
		Simple mode	34	0.051	0.064	0.436	1.052	0.928	1.193				
		Weighted mode	34	-0.009	0.032	0.779	0.991	0.930	1.056				
		MR-PRESSO	34	0.121	0.076	0.121	1.129	0.972	1.310				
**ukb-d-30630_irnt**	Apolipoprotein A	MR Egger	34	0.037	0.041	0.364	1.038	0.959	1.124	0.000	0.150	<0.001	0.791
		Weighted median	34	0.005	0.011	0.670	1.005	0.983	1.026				
		Inverse variance weighted	34	-0.013	0.023	0.573	0.987	0.944	1.032	0.000			
		Simple mode	34	0.001	0.018	0.935	1.001	0.967	1.037				
		Weighted mode	34	0.001	0.010	0.881	1.001	0.983	1.021				
		MR-PRESSO	34	-0.012	0.021	0.575	0.988	0.949	1.030				
**ukb-d-30640_irnt**	Apolipoprotein B	MR Egger	34	0.071	0.042	0.097	1.074	0.990	1.165	0.000	0.637	<0.001	<0.001
		Weighted median	34	0.045	0.010	**0.000**	1.046	1.025	1.067				
		Inverse variance weighted	34	0.055	0.022	**0.015**	1.056	1.011	1.104	0.000			
		Simple mode	34	0.033	0.020	0.096	1.034	0.995	1.074				
		Weighted mode	34	0.040	0.009	**0.000**	1.041	1.022	1.060				
		MR-PRESSO	34	0.051	0.022	**0.024**	1.053	1.009	1.099				
**ukb-d-30790_irnt**	Lipoprotein A	MR Egger	34	0.018	0.055	0.749	1.018	0.913	1.134	0.195	0.713	0.005	0.072
		Weighted median	34	0.017	0.043	0.690	1.017	0.935	1.107				
		Inverse variance	34	0.001	0.030	0.983	1.001	0.944	1.061	0.225			

		Inverse variance weighted											
		Simple mode	34	-0.022	0.073	0.763	0.978	0.847	1.129				
		Weighted mode	34	0.020	0.042	0.628	1.021	0.940	1.108				
		MR-PRESSO	34										
**FinnGen Study**													
**finn-a-I9_K_CARDIAC**	Cardiovascular mortality	MR Egger	31	0.014	0.394	0.971	1.014	0.469	2.194	0.829	0.466	0.486	
		Weighted median	31	-0.114	0.295	0.698	0.892	0.501	1.589				
		Inverse variance weighted	31	-0.232	0.208	0.264	0.793	0.527	1.192	0.842			
		Simple mode	31	-0.128	0.475	0.790	0.880	0.347	2.232				
		Weighted mode	31	0.027	0.321	0.933	1.028	0.548	1.926				
		MR-PRESSO	31	-0.122	0.193	0.533	0.885	0.606	1.294				
**finn-a-I9_MI**	Myocardial infarction	MR Egger	31	0.460	0.456	0.321	1.585	0.648	3.877	0.002	0.338	0.001	0.108
		Weighted median	31	0.296	0.258	0.251	1.345	0.811	2.229				
		Inverse variance weighted	31	0.084	0.242	0.729	1.087	0.677	1.746	0.002			
		Simple mode	31	0.235	0.415	0.575	1.265	0.561	2.853				
		Weighted mode	31	0.325	0.268	0.235	1.383	0.819	2.337				
		MR-PRESSO	31	0.008	0.224	0.970	1.009	0.651	1.563				
**finn-a-I9_ICH**	Intracranial haemorrhage	MR Egger	31	-0.483	0.582	0.413	0.617	0.197	1.931	0.819	0.712	0.741	
		Weighted median	31	-0.348	0.478	0.467	0.706	0.277	1.803				
		Inverse variance weighted	31	-0.299	0.309	0.332	0.742	0.405	1.358	0.847			
		Simple mode	31	-0.909	0.850	0.294	0.403	0.076	2.133				
		Weighted mode	31	-0.328	0.517	0.531	0.721	0.262	1.985				

		MR-PRESSO	31	-0.285	0.263	0.286	0.752	0.449	1.260				
**finn-a-HEARTFAIL**	Heart failure	MR Egger	31	0.110	0.393	0.783	1.116	0.516	2.412	0.012	0.349	0.005	0.173
		Weighted median	31	0.110	0.238	0.644	1.116	0.701	1.778				
		Inverse variance weighted	31	-0.208	0.209	0.319	0.812	0.540	1.222	0.011			
		Simple mode	31	0.265	0.375	0.486	1.303	0.624	2.720				
		Weighted mode	31	0.091	0.246	0.715	1.095	0.676	1.775				
		MR-PRESSO	31	-0.198	0.199	0.327	0.820	0.555	1.212				
**finn-a-I9_ISCHHEART**	Ischemic heart disease	MR Egger	31	0.160	0.363	0.663	1.173	0.576	2.388	0.000	0.853	<0.001	0.151
		Weighted median	31	0.106	0.173	0.540	1.112	0.792	1.562				
		Inverse variance weighted	31	0.102	0.189	0.590	1.108	0.764	1.605	0.000			
		Simple mode	31	0.249	0.292	0.400	1.282	0.724	2.271				
		Weighted mode	31	0.204	0.173	0.249	1.226	0.873	1.722				
		MR-PRESSO	31	0.033	0.179	0.855	1.033	0.728	1.468				
**finn-a-I9_CORATHER**	Coronary atherosclerosis	MR Egger	31	0.250	0.442	0.576	1.284	0.540	3.055	0.000	0.604	<0.001	0.065
		Weighted median	31	0.137	0.205	0.505	1.147	0.767	1.715				
		Inverse variance weighted	31	0.054	0.232	0.817	1.055	0.670	1.662	0.000			
		Simple mode	31	0.267	0.329	0.423	1.306	0.685	2.490				
		Weighted mode	31	0.185	0.197	0.354	1.204	0.818	1.770				
		MR-PRESSO	31	-0.008	0.220	0.970	0.992	0.645	1.525				
**finn-a-I9_CHD**	Major coronary heart disease event	MR Egger	31	0.400	0.402	0.328	1.492	0.679	3.279	0.000	0.412	<0.001	0.739
		Weighted median	31	0.397	0.211	0.060	1.487	0.983	2.249				

		Inverse variance weighted	31	0.116	0.212	0.583	1.123	0.741	1.702	0.000			
		Simple mode	31	0.288	0.339	0.402	1.334	0.687	2.591				
		Weighted mode	31	0.312	0.216	0.158	1.366	0.895	2.085				
		MR-PRESSO	31	0.013	0.205	0.952	1.013	0.678	1.513				
**finn-a-I9_PAD**	Peripheral artery disease	MR Egger	31	0.525	0.550	0.347	1.691	0.576	4.965	0.001	0.424	0.003	0.081
		Weighted median	31	0.241	0.299	0.419	1.273	0.709	2.286				
		Inverse variance weighted	31	0.148	0.290	0.610	1.160	0.657	2.048	0.001			
		Simple mode	31	0.403	0.465	0.393	1.496	0.602	3.718				
		Weighted mode	31	0.294	0.301	0.336	1.342	0.744	2.422				
		MR-PRESSO	31	0.167	0.260	0.525	1.182	0.710	1.966				
**finn-a-I9_HYPTENSESS**	Essential hypertension	MR Egger	31	-0.038	0.473	0.936	0.963	0.381	2.433	0.000	0.701	<0.001	0.422
		Weighted median	31	-0.128	0.191	0.504	0.880	0.605	1.280				
		Inverse variance weighted	31	-0.194	0.248	0.435	0.824	0.507	1.339	0.000			
		Simple mode	31	-0.064	0.293	0.827	0.938	0.528	1.664				
		Weighted mode	31	-0.090	0.199	0.656	0.914	0.619	1.351				
		MR-PRESSO	31	-0.216	0.238	0.371	0.806	0.505	1.286				
**finn-a-C_STROKE**	Stroke	MR Egger	31	-0.423	0.424	0.326	0.655	0.285	1.503	0.000	0.451	<0.001	0.487
		Weighted median	31	-0.316	0.242	0.192	0.729	0.453	1.172				
		Inverse variance weighted	31	-0.149	0.224	0.506	0.862	0.555	1.336	0.000			
		Simple mode	31	0.649	0.522	0.223	1.914	0.688	5.318				
		Weighted mode	31	-0.452	0.216	**0.045**	0.637	0.417	0.972				

		MR-PRESSO	31	-0.091	0.203	0.656	0.913	0.613	1.360				
**finn-a-I9_STR_EXH**	Ischemic stroke	MR Egger	31	-0.321	0.418	0.450	0.726	0.320	1.647	0.008	0.803	0.010	0.279
		Weighted median	31	-0.345	0.276	0.211	0.708	0.412	1.216				
		Inverse variance weighted	31	-0.231	0.219	0.290	0.794	0.517	1.218	0.011			
		Simple mode	31	-0.451	0.470	0.345	0.637	0.253	1.602				
		Weighted mode	31	-0.403	0.268	0.143	0.668	0.395	1.130				
		MR-PRESSO	31	-0.136	0.196	0.492	0.872	0.594	1.282				
**finn-a-I9_AF**	Atrial fibrillation and fluttering	MR Egger	31	0.244	0.515	0.639	1.277	0.465	3.505	0.000	0.193	<0.001	0.84
		Weighted median	31	0.211	0.253	0.403	1.235	0.753	2.026				
		Inverse variance weighted	31	-0.337	0.277	0.224	0.714	0.415	1.230	0.000			
		Simple mode	31	0.342	0.449	0.452	1.407	0.584	3.392				
		Weighted mode	31	0.195	0.248	0.437	1.216	0.748	1.975				
		MR-PRESSO	31	-0.405	0.261	0.130	0.667	0.400	1.113				
**The Global Lipids Genetics Consortium**													
**ieu-a-299**	HDL cholesterol	MR Egger	33	0.248	0.191	0.205	1.281	0.880	1.864	0.000	0.266	<0.001	0.584
		Weighted median	33	0.148	0.056	**0.008**	1.160	1.039	1.294				
		Inverse variance weighted	33	0.059	0.096	0.534	1.061	0.880	1.280	0.000			
		Simple mode	33	0.137	0.075	0.077	1.147	0.990	1.328				
		Weighted mode	33	0.148	0.056	**0.012**	1.160	1.040	1.293				
		MR-PRESSO	33	0.040	0.091	0.662	1.041	0.871	1.243				
**ieu-a-300**	LDL cholesterol	MR Egger	33	0.245	0.323	0.453	1.278	0.679	2.408	0.000	0.969	<0.001	0.748

		Weighted median	33	0.185	0.069	**0.007**	1.204	1.052	1.378					
		Inverse variance weighted	33	0.234	0.158	0.137	1.264	0.928	1.721	0.000				
		Simple mode	33	0.062	0.118	0.602	1.064	0.844	1.341					
		Weighted mode	33	0.146	0.072	0.052	1.157	1.004	1.333					
		MR-PRESSO	33	0.188	0.142	0.196	1.206	0.913	1.595					
**ieu-a-302**	Triglycerides	MR Egger	33	-0.235	0.145	0.117	0.791	0.595	1.052	0.000		0.121	<0.001	0.314
		Weighted median	33	-0.109	0.059	0.062	0.896	0.799	1.006					
		Inverse variance weighted	33	-0.034	0.075	0.646	0.966	0.834	1.119	0.000				
		Simple mode	33	-0.066	0.099	0.512	0.936	0.771	1.138					
		Weighted mode	33	-0.104	0.060	0.095	0.902	0.801	1.014					
		MR-PRESSO	33	0.014	0.076	0.853	1.014	0.874	1.178					

*No significant outliers

Table S8 HDL-C as a mediator in the causal pathway between T2DM and risk of CVDs

Outcome	Total effect	Direct effect	Mediation effect	Mediation proportion (95% CI)
Cardiovascular mortality	0.056	0.049	0.0071	12.8% (-0.4%, 39.3%)
**Myocardial infarction**	0.134	0.138	-0.0044	**7.4% (0.8%, 20.6%)**
Intracerebral hemorrhage	0.078	0.077	0.0003	0.4% (-9.2%, 29.0%)
Ischemic heart disease	0.114	0.113	0.0003	0.2% (-6.3%, 19.1%)
**Coronary atherosclerosis**	0.118	0.103	0.0152	**12.8% (2.4%, 31.0%)**
Major coronary heart disease event	0.103	0.089	0.0139	13.5% (2.4%, 33.2%)
**Peripheral artery disease**	0.183	0.164	0.0195	**10.6% (2.4%, 24.6%)**
**Essential hypertension**	0.122	0.115	0.0072	**5.9% (0.2%, 17.3%)**
Stroke	0.099	0.093	0.006	6.1% (-0.5%, 19.6%)
Ischemic stroke	0.063	0.057	0.0052	8.4% (-1.8%, 30.2%)

Table S9 Triglycerides as a mediator in the causal pathway between T2DM and risk of CVDs

Outcome	Total effect	Direct effect	Mediation effect	Mediation proportion (95% CI)

Cardiovascular mortality	0.056	0.056	0.0004	0.7% (-1.9%, 24.2%)
**Myocardial infarction**	0.134	0.118	0.0158	**11.9% (1.1%, 31.2%)**
Intracerebral hemorrhage	0.078	0.075	0.0026	3.3% (-1.9%, 35.0%)
Ischemic heart disease	0.114	0.105	0.0084	7.4% (-19.0%, 53.0%)
Coronary atherosclerosis	0.118	0.108	0.0100	8.5% (-21.6%, 60.5%)
Major coronary heart disease event	0.103	0.093	0.0095	9.2% (-6.2%, 35.5%)
**Peripheral artery disease**	0.183	0.171	0.0122	**6.7% (0.2%, 21.6%)**
**Essential hypertension**	0.122	0.113	0.0089	**7.3% (0.4%, 22.2%)**
Stroke	0.099	0.095	0.0033	3.4% (-0.3%, 15.8%)
Ischemic stroke	0.063	0.064	-0.0013	-2.0% (-1.9%, 15.3%)

Table S10 ApoA as a mediator in the causal pathway between T2DM and risk of CVDs

Outcome	Total effect	Direct effect	Mediation effect	Mediation proportion (95% CI)
Cardiovascular mortality	0.056	0.054	0.0017	3.0% (-5.9%, 18.3%)
**Myocardial infarction**	0.134	0.125	0.0088	**6.6% (1.2%, 14.9%)**
Intracerebral hemorrhage	0.078	0.081	-0.0038	-4.9% (-14.0%, 12.8%)
**Ischemic heart disease**	0.114	0.104	0.0097	**8.5% (2.3%, 17.8%)**
**Coronary atherosclerosis**	0.118	0.108	0.0104	**8.8% (2.2%, 18.8%)**
**Major coronary heart disease event**	0.103	0.093	0.0103	**10.0% (2.6%, 21.0%)**
**Peripheral artery disease**	0.183	0.173	0.01	**5.5% (1.0%, 12.3%)**
Essential hypertension	0.122	0.121	0.0016	1.3% (-1.9%, 6.8%)
**Stroke**	0.099	0.092	0.0064	**6.5% (1.0%, 14.9%)**
Ischemic stroke	0.063	0.057	0.0054	8.5% (-0.2%, 22.5%)

Table S11 Multivariable Mendelian randomization using IVW method.

**Outcome**	**Exposure**	**No. of SNPs**	**Beta**	**SE**	**OR**	**LCI**	**UCI**	***p* value**
**Cardiovascular mortality**	T2DM	80	0.001	0.000	1.001	1.000	1.001	**0.031**
	HbA_{1C}	6	0.000	0.001	1.000	0.997	1.002	0.779
	FG	27	0.003	0.001	1.003	1.001	1.005	**0.006**
	FI	11	0.002	0.002	1.002	0.997	1.007	0.374
**Myocardial infarction**	T2DM	80	0.135	0.038	1.144	1.062	1.233	**0.000**
	HbA_{1C}	6	0.193	0.244	1.213	0.752	1.957	0.428
	FG	27	-0.088	0.174	0.916	0.651	1.289	0.615
	FI	11	0.109	0.311	1.115	0.606	2.052	0.727

**Intracranial haemorrhage**	T2DM	80	0.000	0.000	1.000	0.999	1.000	0.707
	HbA_{1C}	6	0.001	0.002	1.001	0.998	1.004	0.606
	FG	27	-0.002	0.001	0.998	0.996	1.000	0.107
	FI	11	0.002	0.002	1.002	0.998	1.007	0.351
**Heart failure**	T2DM	75	0.037	0.023	1.037	0.991	1.086	0.114
	HbA_{1C}	5	-0.181	0.142	0.835	0.631	1.104	0.205
	FG	26	0.206	0.115	1.228	0.980	1.540	0.075
	FI	11	-0.229	0.188	0.795	0.550	1.150	0.224
**Ischemic heart disease**	T2DM	80	0.160	0.038	1.173	1.089	1.263	**0.000**
	HbA_{1C}	6	0.119	0.242	1.127	0.702	1.809	0.621
	FG	27	-0.034	0.172	0.966	0.689	1.354	0.842
	FI	11	0.248	0.308	1.281	0.701	2.343	0.421
**Coronary atherosclerosis**	T2DM	80	0.006	0.002	1.006	1.002	1.009	**0.002**
	HbA_{1C}	6	0.011	0.011	1.011	0.990	1.033	0.310
	FG	27	0.003	0.008	1.003	0.987	1.019	0.698
	FI	11	0.002	0.015	1.002	0.973	1.032	0.902
**Major coronary heart disease event**	T2DM	80	0.006	0.002	1.006	1.002	1.009	**0.002**
	HbA_{1C}	6	0.011	0.011	1.011	0.990	1.033	0.310
	FG	27	0.003	0.008	1.003	0.987	1.019	0.698
	FI	11	0.002	0.015	1.002	0.973	1.032	0.902
**Peripheral artery disease**	T2DM	75	0.300	0.067	1.350	1.184	1.540	**0.000**
	HbA_{1C}	4	0.537	0.446	1.712	0.714	4.102	0.228
	FG	25	-0.332	0.348	0.717	0.363	1.418	0.339
	FI	11	1.092	0.496	2.980	1.128	7.871	**0.028**
**Essential hypertension**	T2DM	80	0.013	0.002	1.013	1.009	1.017	**0.000**
	HbA_{1C}	6	0.015	0.013	1.015	0.989	1.042	0.250
	FG	27	-0.018	0.010	0.982	0.963	1.001	0.063
	FI	11	0.071	0.018	1.074	1.036	1.113	**0.000**
**Stroke**	T2DM	80	0.122	0.022	1.129	1.082	1.179	**0.000**

	HbA_{1C}	6	-0.125	0.134	0.882	0.678	1.148	0.351
	FG	27	-0.054	0.099	0.948	0.781	1.151	0.588
	FI	11	-0.031	0.178	0.969	0.683	1.374	0.861
**Ischemic stroke**	T2DM	80	0.130	0.022	1.139	1.090	1.190	**0.000**
	HbA_{1C}	6	-0.176	0.136	0.838	0.642	1.095	0.196
	FG	27	-0.011	0.102	0.989	0.809	1.208	0.911
	FI	11	-0.121	0.183	0.886	0.619	1.269	0.509
**Atrial fibrillation and fluttering**	T2DM	80	0.003	0.022	1.003	0.961	1.047	0.891
	HbA_{1C}	6	-0.079	0.136	0.924	0.707	1.208	0.564
	FG	27	0.023	0.099	1.023	0.842	1.243	0.818
	FI	11	-0.173	0.180	0.841	0.591	1.197	0.336
**HDL cholesterol**	T2DM	80	-0.041	0.010	0.960	0.941	0.979	**0.000**
	HbA_{1C}	6	0.065	0.062	1.067	0.946	1.204	0.290
	FG	27	0.028	0.045	1.029	0.941	1.124	0.534
	FI	11	-0.397	0.084	0.673	0.570	0.793	**0.000**
**LDL cholesterol**	T2DM	80	-0.036	0.015	0.965	0.936	0.994	**0.017**
	HbA_{1C}	6	0.219	0.092	1.244	1.040	1.489	**0.017**
	FG	27	-0.089	0.068	0.915	0.802	1.044	0.188
	FI	11	-0.198	0.125	0.820	0.642	1.048	0.113
**Triglycerides**	T2DM	80	0.090	0.037	1.094	1.018	1.175	**0.014**
	HbA_{1C}	6	0.079	0.222	1.083	0.701	1.673	0.720
	FG	27	-0.250	0.164	0.779	0.565	1.074	0.128
	FI	11	0.197	0.304	1.218	0.671	2.212	0.516
**Apolipoprotein A**	T2DM	80	-0.021	0.006	0.979	0.968	0.990	**0.000**
	HbA_{1C}	6	0.042	0.035	1.043	0.974	1.117	0.227

	FG	27	-0.005	0.026	0.995	0.946	1.046	0.831
	FI	11	-0.278	0.048	0.758	0.690	0.832	**0.000**
**Apolipoprotein B**	T2DM	80	-0.005	0.005	0.995	0.986	1.005	0.301
	HbA_{1C}	6	0.059	0.029	1.061	1.001	1.124	**0.046**
	FG	27	-0.025	0.022	0.975	0.935	1.018	0.248
	FI	11	-0.030	0.040	0.970	0.897	1.050	0.453
**Lipoprotein A**	T2DM	80	0.013	0.047	1.013	0.924	1.110	0.781
	HbA_{1C}	6	0.363	0.283	1.437	0.825	2.502	0.200
	FG	27	-0.184	0.209	0.832	0.552	1.254	0.380
	FI	11	-0.616	0.388	0.540	0.252	1.156	0.112
